# Hot and Hungry - High temperatures induce changes in leaf carbon dynamics and sugar isotope fingerprints

**DOI:** 10.1038/s44383-025-00012-6

**Published:** 2025-12-01

**Authors:** Philipp Schuler, Margaux Didion-Gency, Valentina Vitali, Matthias Saurer, Manuela Oettli, Haoyu Diao, Nina Buchmann, Arthur Gessler, Marco M. Lehmann

**Affiliations:** 1 Forest and Soil Ecology, Swiss Federal Institute for Forest, Snow and Landscape Research WSL, Birmensdorf, Switzerland; 2 Department of Environmental Systems Science, ETH Zurich, Zurich, Switzerland; 3 Plant Ecology Research Laboratory PERL, School of Architecture, Civil and Environmental Engineering ENAC, EPFL, Lausanne, Switzerland; 4 Ecological and Forestry Applications Research Center (CREAF), Cerdanyola del Valley, Spain; 5 Forest Ecology, Institute of Terrestrial Ecosystems, Department of Environmental Systems Science, ETH Zürich, Zürich, Switzerland

**Keywords:** Environmental sciences, Plant physiology, Heat

## Abstract

Predicting vegetation responses to global warming and reconstructing them from stable isotope records requires a clear understanding of how they respond to temperature. We investigated leaf carbon-dynamics (gas exchange, non-structural carbohydrate (NSC)) and the hydrogen (δ²H) and oxygen (δ¹⁸O) isotopic composition of leaf water and sugars in well-watered C₃ trees, forbs, grasses, and one C₄ grass across air temperatures from 10 °C to 40 °C at a low VPD. In C₃ species, temperatures ≥30 °C reduced A_net_, increased R_dark_, and shifted NSC composition from starch to sugars. Concurrently, apparent δ²H and δ¹⁸O fractionation between leaf water and sugars decreased. δ²H and δ¹⁸O in C₃ plants can be modeled from leaf water isotope composition when accounting for temperature-driven changes in gas exchange and NSC dynamics. These findings reveal heat-induced shifts in carbohydrate metabolism leave distinct isotope signatures, providing a mechanistic basis for improved isotope models to study current and past tree carbon dynamics.

## Introduction

Temperature is a key driver of leaf physiological responses in plants. However, under natural conditions, its effects are often confounded by vapor pressure deficit, and technical challenges make it difficult to isolate the impact of temperature, particularly at extreme levels (e.g., >30 °C). As a result, how specific temperature impacts leaf level carbon dynamics (i.e., photosynthesis, respiration, carbohydrate metabolism) and the resulting isotope patterns remain not fully understood. Here we aimed to isoloate the temperature effect (10 to 40 °C in 5 °C steps) for a wide range of species from different plant functional types (i.e., trees, grasses, forbs) to improve the mechanistic understanding on the dynamics of leaf physiology and its impact on hydrogen (δ^2^H) and oxygen (δ^18^O) isotope pattern in plant carbohydrates.

The analysis of stable isotope ratios is a widely applied tool to reconstruct past and study current responses of plants to various environmental drivers^1–3^. While traditional mechanistic models to predict δ^2^H and δ^18^O in tree ring cellulose^4^ indeed separate photosynthetic (ε_A_, i.e., autotrophic) and post-photosynthetic ε_H_, i.e., heterotrophic) fractionation processes, they have traditionally been considered to be constant within and among species and in response to environmental drivers^2,5^. However, recent research has shown that the fractionation of oxygen and especially hydrogen isotopes being highly variabile among different photosynthetic types (i.e., C_3_, C_4_, CAM), with up to 20‰ in δ^18^O and more than 200‰ in δ^2^H^6,7^. The observed high variability in δ^2^H mostly derives from the large variability within C_3_ species^8,9^. Furthermore, the ^2^H fractionation is strongly responding to environmental drivers^10–12^, but even δ^18^O is known to have a distinct temperature response^13^. Deriving from the available source water, primary assimilates such as sucrose form the base of the isotopic composition of cellulose of leaves^14^ and twig xylem^4,8^. Therefore, to fully understand the observed δ^2^H and δ^18^O in tree-ring cellulose, it is crucial to understand how the observed apparent autotrophic ^2^H and ^18^O fractionations are responding to environmental drivers. It has been shown that the temperature effect of δ^18^O in tree-ring cellulose is closely correlated to the vapor pressure deficit (VPD) of the air^15^, however, to our knowledge, no study isolated the pure temperature effect on the authotrophic ^18^O fractionation from leaf water to leaf sugar. The fractionation of ^2^H and ^18^O during NSC synthesis and metabolism is influenced by drivers such as temperature^13^, and different biochemical reactions^7,16,17^. Recent studies suggest that particular the hydrogen isotope composition of plant carbohydrates strongly responds to changes in leaf gas exchange and NSC pools^10^, and are connected to leaf dark respiration (R_dark_)^14^. Other recent studies show the possible link between a negative plant carbon balance and a ^2^H enrichment in plant organic matter^18^, and a decoupling between δ^2^H and δ^18^O in tree-ring cellulose delivered important information whether the carbon balance of a tree is out of balance^11,12^. However, these studies fall short of providing enough physiologically relevant measurements with high resolution under changing environmental conditions to investigate how leaf gas exchange and carbohydrate metabolism are integrated into the stable isotope pattern of plant carbohydrates. Therefore, there is still a lack of comprehensive studies directly connecting the temperature response of plant physiological processes, carbohydrate metabolism, and the fractionation of ^2^H and ^18^O in leaf sugar. To overcome this limitation, we investigate and link the temperature responses of leaf internal carbon dynamics with the apparent photosynthetic ^2^H and ^18^O isotope fractionation.

Photoautotrophic CO_2_ fixation, one key process in the global carbon cycle and the primary process that provides the carbohydrates needed for plant growth and productivity, is highly dependent on several environmental factors, of which temperature is one of the most important^19^. The optimum temperature for the net CO_2_ assimilation (A_net_) varies between plant species, typically ranging from 20–30 °C, usually align with the prevailing growth temperatures in their environment^20^, and can be stable until 46 °C in plant species adapted to heat^21^. Above the optimum temperature, A_net_ decreases due to several processes, including damage to the photosynthetic machinery^22–24^. Post-photosynthetic metabolic processes such as mitochondrial respiration provide energy for growth and maintenance, and are thus essential for plant reproduction and survival. Respiratory processes continue to increase until much higher temperatures than photosynthesis^25^ due to increased enzyme activity^26^ and maintenance costs^27^. This can lead to a decrease in energy efficiency if maintenance costs become too high, thereby reducing the plant’s ability to produce a surplus of carbohydrates for growth and storage^28^.

Since A_net_ and respiration are temperature-dependent, by extension plant non-structural carbohydrate (NSC, i.e., sugar, starch) dynamics are also temperature-dependent^29–31^. Assessing the effects of rising temperatures on plant metabolism can be challenging because temperature changes are often associated with concomitant changes in vapor pressure deficit (VPD)^32,33^, which can impact plant physiology and biochemistry in complex ways^34,35^. For instance, high VPD can lead to a reduction in stomatal conductance (g_s_) and therefore CO_2_ uptake^36^, affecting A_net_^37^ and, ultimately, plant metabolism and growth^38^. However, experiments aiming to isolate temperature from VPD effects on leaf gas exchange and NSC metabolism at plant physiologically relevant temperature ranges are still rare due to technical challenges^33,39,40^.

To isolate the effects of rising temperature under constant VPD on plant metabolism, we conducted a climate chamber experiment where we grew six C_3_ (including two trees, *Quercus pubescens* Willd. and *Phytolacca dioica* L.; two grasses, *Hordeum vulgare* L. and *Oryza sativa* L.; and two forbs, *Salvia hispanica* L. and *Solanum cheesmaniae* (Riley) Fosburg) and one C_4_ (*Sorghum bicolor* (L.) Moench) plant species. As shown by Schuler et al.^7^, C_4_ plants, unlike C_3_ plants, did not show a temperature response in their δ^2^H of leaf sugar and leaf cellulose, and was therefore used as a control. This selection includes one temperate and one subtropical tree species and five agriculturally important crops from different geographical and climatic zones. Similar to Wieloch et al.^17^, we reduced the old NSC pool between each temperature cycle by keeping the plants in the dark at 20 °C for 48 h. This reduction of old NSC was used to ensure that the measured δ^2^H and δ^18^O in sugars corresponded to the physiological conditions of the plants at each specific temperature. We exposed the plants to a constant daytime temperature for five days for 18 h each, starting at 10 °C and subsequently increasing to 40 °C in 5 °C steps. This allowed the plants to acclimate photosynthesis and respiration^41^ to each tested temperature. Water supply (kept at field capacity), light (PAR = 800 µmol m^−2^ s^−1^ during the daytime hours), and VPD (1 kPa, through regulation of relative humidity) were kept constant. On the fourth day, we sampled leaves for non-structural carbohydrates (NSC) and stable isotope (δ^18^O and δ^2^H of leaf sugars and water) analyses. On the fifth day, we measured leaf gas exchange (A_net_, R_dark_, g_s_) and chlorophyll fluorescence. The gained knowledge will help to better understand the measured oxygen and hydrogen isotope fractionations in plant carbohydrates and to eventually update current isotope models^4^.

## Results

### Temperature response of the leaf gas exchange and the electron transport rate in PSII

**Fig. 1:**
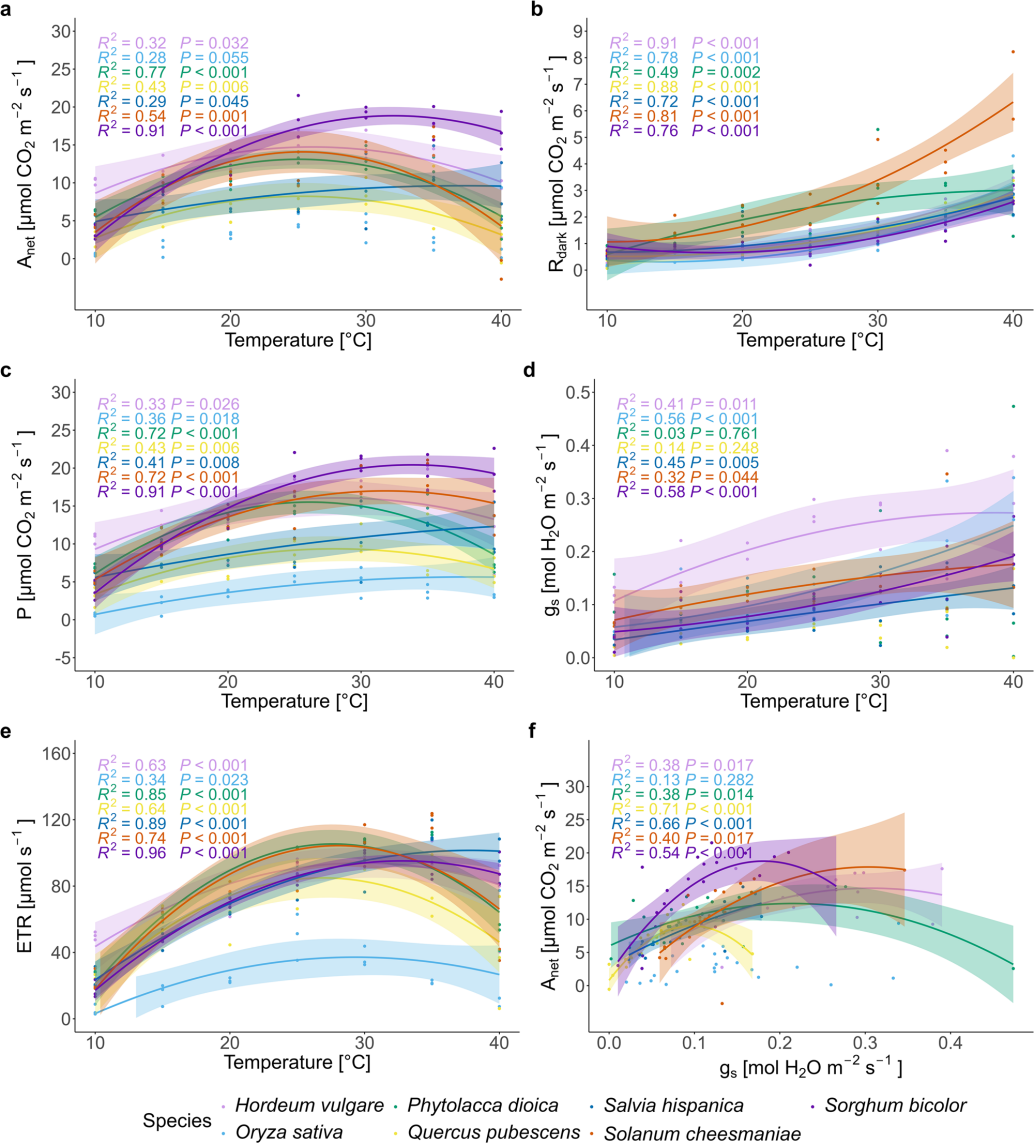
Temperature response of leaf gas exchange, electron transport rate, and stomatal decoupling. Species-specific temperature response of **a** the net assimilation rate (A_net_), **b** dark respiration rates (R_dark_), **c** gross photosynthesis (P, i.e., A_net_ + R_dark_), **d** stomatal conductance (g_s_), **e** electron transport rates (ETR), and **f** the relationship between A_net_ and g_s_. Colors indicate species, the quadratic models depicting the relationship are shown only for species with a significant temperature response (*p* ≤ 0.05), and the light shading denotes the 95% confidence level interval for predictions of the quadratic fit.

Leaf physiology responded strongly to the temperature treatment from 10 to 40 °C (Fig. 1, Supplementary Tables 1 and 2). A_net_ (Fig. 1a) of the C_3_ species, except *Salvia hispanica*, reached its maximum between 25 and 30 °C, followed by a steady decline with further temperature increase. On the other hand, A_net_ of the C_3_ plant *S. hispanica* and the C_4_ plant *Sorghum bicolor* increased until 30 °C and remained largely stable until 40 °C. Leaf dark respiration (R_dark_, Fig. 1b) increased strongest in *Solanum cheesmaniae* with increasing temperature, while R_dark_ of *P. dioica* was in general higher between 15 and 35 °C compared to the other species, which exhibited similar and modest increases with rising temperatures. Gross photosynthesis (P, A_net_ + R_dark_, Fig. 1c) increased with increasing temperature in all species but decreased strongly only in *Phytolacca dioica* above 30 °C. Stomatal conductance (g_s_, Fig. 1d), while being highly variable among all tested species, as well as its response to increasing temperatures, generally increased with higher temperatures. While *S. hispanica* and *S. bicolor* maintained a stable electron transport rate (ETR, Fig. 1e) above 25 °C, ETR of the other C_3_ species showed a strong decrease above 30 °C (ETR measurements of C_4_ species does not give reliable results with the used method). A_net_ was significantly related to g_s_ in five of the seven species (Fig. 1f), but since g_s_ kept increasing after A_net_ reached its optimum, their relationship decoupled or reversed (i.e., became negative) at high temperatures^40^.

### Changes in non-structural carbohydrate concentration and composition in response to rising temperatures

**Fig. 2:**
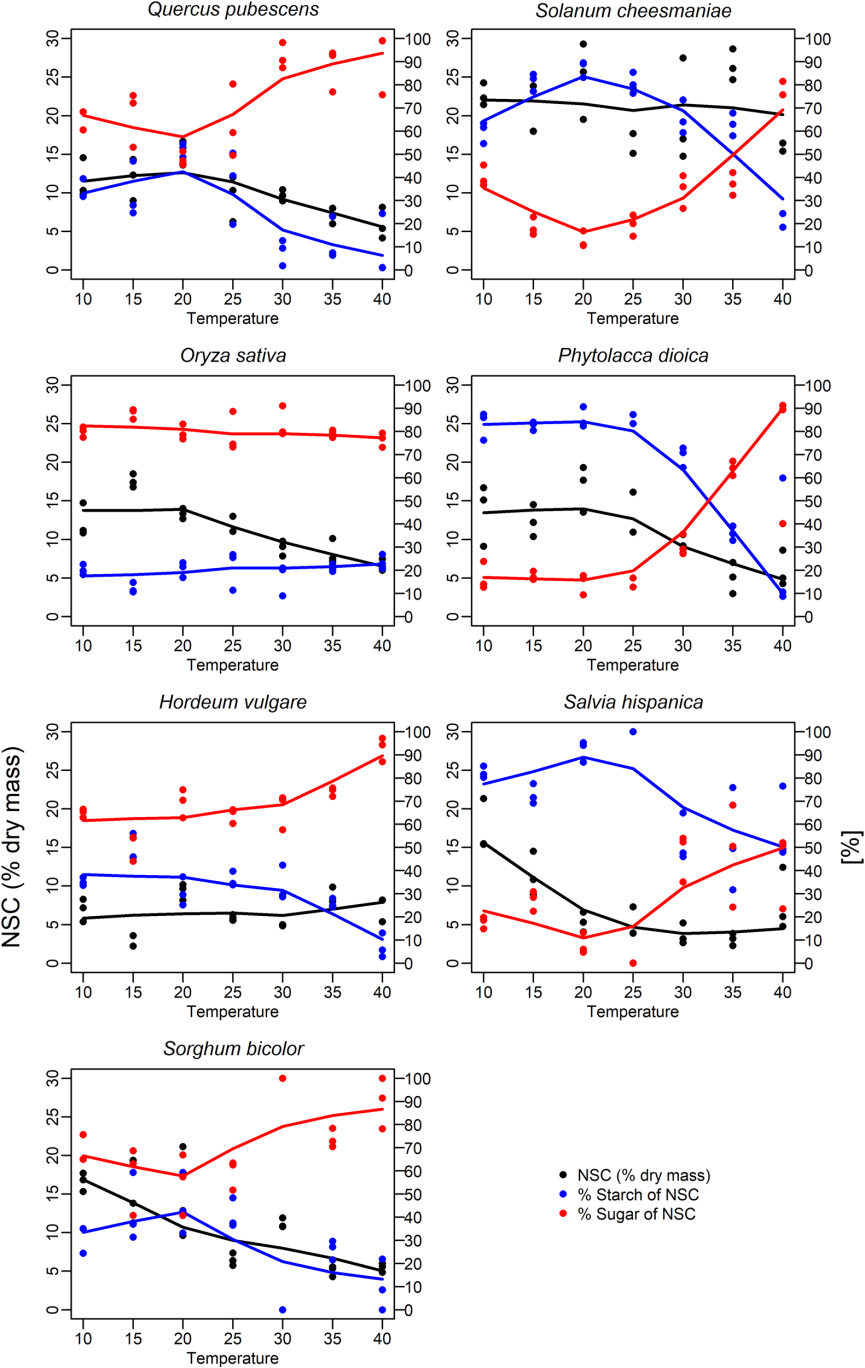
Temperature response of non-structural carbohydrates in leaves. Runing mean of species-specific temperature response of the total content of non-structural carbohydrates (NSC in % of leaf dry mass, black, left y-axis), and the percentage of the leaf NSC pools consisting of either starch (% Starch, blue, right y-axis) or sugar (% Sugar, red). Each variable is smoothed using lowess (locally weighted scatterplot smoothing) to show trends across the temperature range.

The concentration and composition of leaf NSC responded significantly to temperature (Fig. 2, Supplementary Tables 3 and 4). Across all species, the total NSC concentration was significantly higher at lower temperatures (10–15 °C) compared to higher temperatures (35–40 °C) (Mann–Whitney U test *p* < 0.001), with an average of 13.72% of the leaf dry mass at lower temperatures *vs*. 7.84% at higher temperatures. Similarly, the percentage of starch that contributes to the total leaf NSC pool was significantly higher at lower temperatures (51.44%) than at higher temperatures (21.96%) (Mann-Whitney U test *p* < 0.001). Two groups can be identified based on their NSC storage strategy: One group consisting of the temperate tree and the grass species (Fig. 2 left panels; *Quercus pubescens*, *Oryza sativa, Hordeum vulgare*, and *S. bicolor)* always stored most of their total NSC pool as sugar. In contrast, the subtropical tree and forbs (Fig. 2, right panels; *S. cheesmaniae*, *P. dioica*, and *S. hispanica*) stored most of the total leaf NSC as starch at moderate temperatures. Lastly, *O. sativa* was the only species with no significant change in its NSC composition, with 80% of the leaf NSC stored as sugar at all temperatures.

### The dual isotope response to rising temperature

**Fig. 3:**
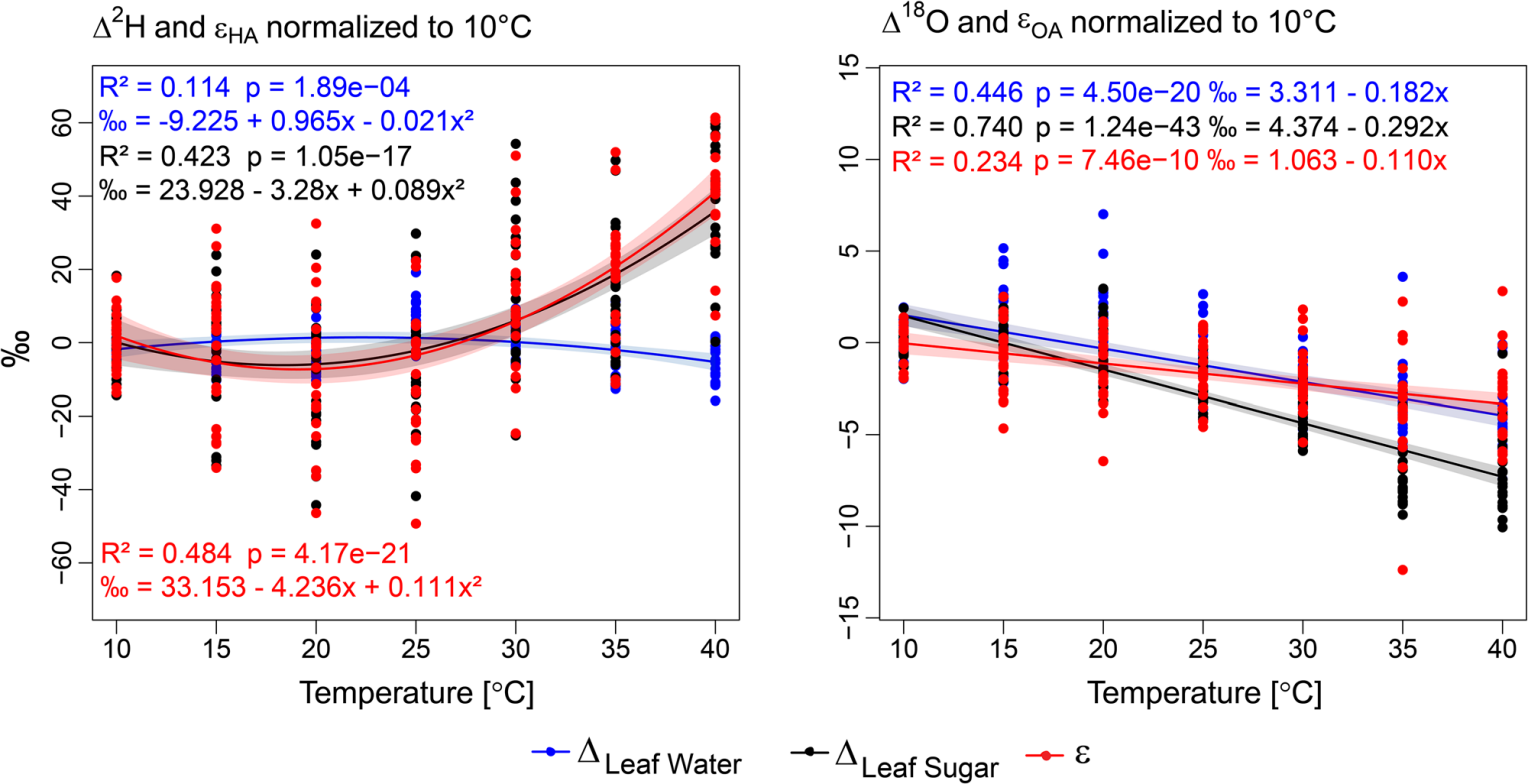
The temperature response of ^2^H and ^18^O. The isotope response to temperature of Δ^2^H and Δ^18^O in leaf water and leaf sugar, and ε_HA_ and ε_OA_, the apparent ^2^H and ^18^O fractionation between leaf water and leaf sugar. Values are given as Δ; e.g., normalized to average δ values measured at 10 °C individually for each species. The lines represent the quadratic (^2^H) respectively the linear model (^18^O) depicting the relationship between temperature and Δ and ε values, the lighter shading denotes the 95% confidence level interval for predictions of the quadratic and linear fit, R^2^, and p values as well as the corresponding functions for the temperature response (i.e., ‰ at a given temperature) values inform on the temperature relationship of the variables.

Across all species, the two measured isotope ratios (δ^18^O and δ^2^H) showed distinct responses to the increasing temperature (Fig. 3, Supplementary Figs. 2, 3, Supplementary Table 5). While the observed temperature effect on Δ^2^H of leaf water (Δ values are normalized to the initial average value for each species at 10 °C) was significant but small (R^2^ = 0.114, *p* < 0.01), higher temperature led to a non-linear increase in Δ^2^H of leaf sugar (R^2^ = 0.423, *p* < 0.001), which was caused by the temperature response of the biological ^2^H fractionation ε_HA_ (R^2^ = 0.484, *p* < 0.001). In contrast, Δ^18^O of the leaf sugar decreased linearly with increasing temperature (R^2^ = 0.74, *p* < 0.001), reflecting a combination of the linear decreases in leaf water Δ^18^O and ε_OA_ (R^2^ = 0.446, *p* < 0.001 and R^2^ = 0.234, *p* < 0.001, respectively), leading to an overall decrease of ε_OA_ by 0.11‰ per 1 °C temperature increase. We found a positive relationship (covariation) between Δ^18^O and Δ^2^H in leaf water within and across all tested temperatures (Supplementary Fig. 4). However, in leaf sugar, we only found a significant and positive relationship between Δ^18^O and Δ^2^H at 25 and 30 °C, while the overall relationship across all temperatures was negative (Supplementary Fig. 4).

### Exploring the drivers of leaf sugar δ^2^H and δ^18^O by using generalized additive models (GAM)

The 34 GAM evaluated for δ^2^H (Supplementary Table 6) and 19 GAM evaluated for δ^18^O (Supplementary Table 7) of δ^2^H and δ^18^O of leaf sugar (δ^2^H_LS_, δ^18^O_LS_) showed a wide range of explanatory power. For δ^2^H of leaf sugar (δ^2^H_LS_), the species-specific average apparent fractionation between leaf water and leaf sugar (ε_HA25_), representing the baseline in all models, explained 58.8% of the variation (model 34: adj. R^2^ = 0.581, *p* < 0.001). The explanatory power of δ^2^H of leaf water (δ^2^H_LW_) for δ^2^H_LS_ was only significant for two simple versions of the δ^2^H_LS_ model (model 33: adj. R^2^ = 0.612, *p* < 0.01). However, as δ^2^H_LW_ forms the basis of the isotopic composition of δ^2^H_LS_, it is expected to have a significant and consistent effect over larger geographic or temporal scales when the δ^2^H value of the source water differs considerably. Thus, the δ^2^H_LW_ was not removed in the more complex models, although its contribution was not significant in this study, it will be necessary to integrate δ^2^H of the source water into the model to predict leaf sugar δ^2^H. Assimilation (A_net_) and dark respiration (R_dark_) were found to have a significant impact on the explanatory power of the models (model 32: adj. R^2^ = 0.631, *p* < 0.001, and model 29: adj. R^2^ = 0.657, *p* < 0.001, respectively), and an even greater impact when considered in interaction with each other (model 23: adj. R^2^ = 0.722, *p* < 0.001). Including the NSC concentration (% dry mass; model 31: adj. R^2^ = 0.655, *p* < 0.001) as well as the percentage starch contributes to the total NSC pool (% of NSC; model 22: adj. R^2^ = 0.726, *p* < 0.001) also significantly contributed to the model performance. The best model combined the interactive terms of temperature with R_dark_ (<0.01), temperature with A_net_ (<0.001), and the interaction between the NSC concentration and the percentage starch contributes to the total NSC pool (<0.001) and explained 90.7% of the variation (model 1: adj. R^2^ = 0.884).

**Fig. 4:**
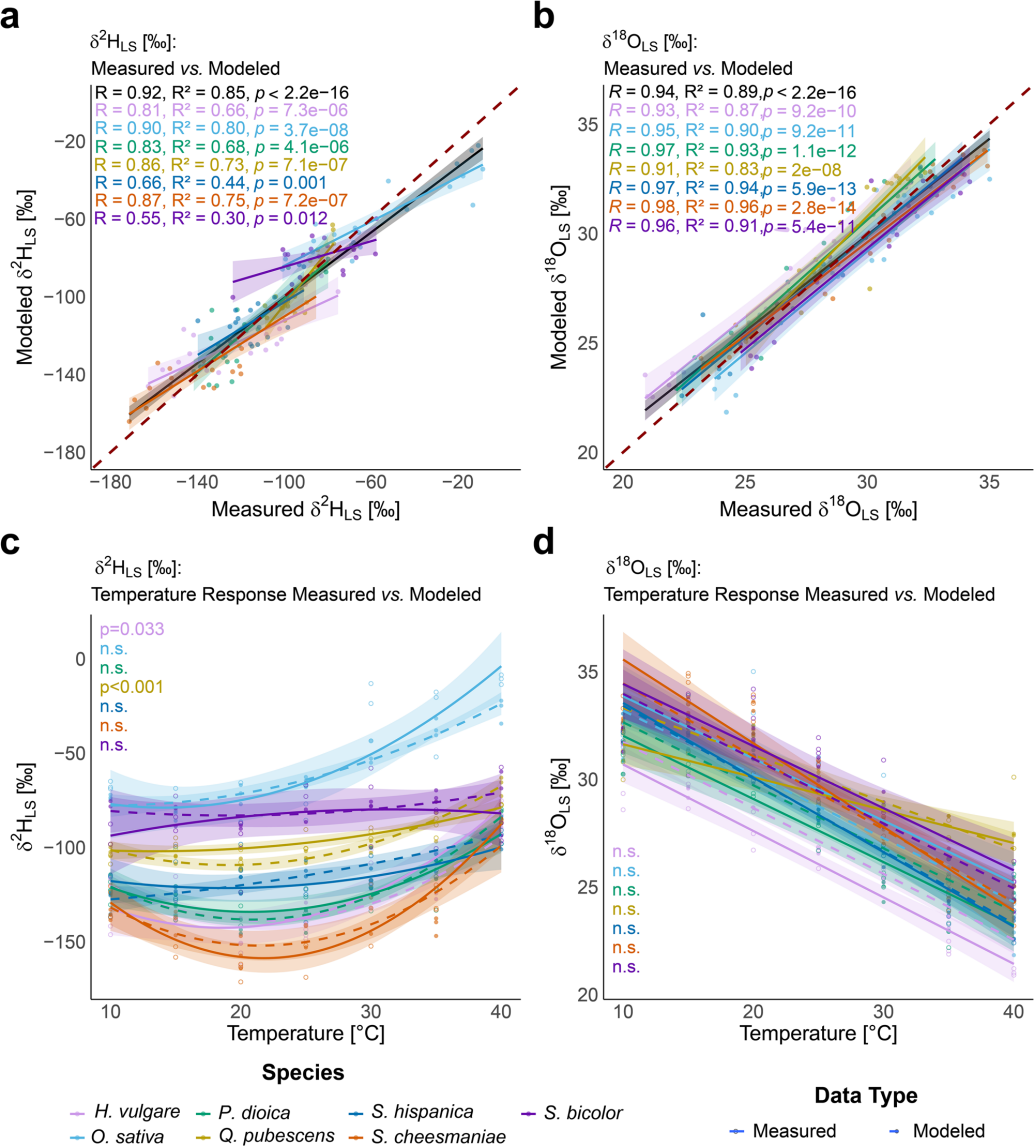
Modeled vs. measured ^2^H and ^18^O fractionation. Comparison and evaluations of the modeled *vs*. the measured δ^2^H_LS_ (**a**, **c**) and δ^18^O_LS_ (**b**, **d**), using the newly developed generalized additive model (GAM). **a**, **b** Scatter plots of the measured vs. the modeled δ^2^H_LS_ and δ^18^O_LS_, respectively. The linear regression line (violet) with the 95% confidence intervals (shaded in pink), illustrates the relationship between measured and modeled values, as well as their R, R^2^, and *p*-values. The dashed 1:1 line is a reference to assess model performance visually. **c**, **d** Temperature response of the modeled (dashed line) and measured (straight line) δ²H_LS_ and δ^18^O_LS_, respectively, including significance analysis between the predicted and observed temperature response (i.e., n.s. when the precited temperature response did not significantly differ from observed response).

Plotting the modeled data of model 1 against the measured δ^2^H_LS_ (Fig. 4a) further confirmed the largely good reproducibility of the measured data (R = 0.95, *p* < 0.001, R^2^ = 0.91, m = 0.9). Furthermore, the model slightly overestimates the lowest and underestimates the highest δ^2^H_LS_. However, the model can reproduce the observed δ^2^H_LS_ temperature response well (Fig. 4b; RMSE = 10.13, Mean Absolute Error (MAE) = 8.12).

δ^18^O_LW_ was the baseline for the 19 evaluated GAM for δ^18^O_LS_ (Supplementary Table 7) and explained 64.3% of the observed variation (adj. R^2^ = 0.634, *p* < 0.001). Again, the best model (adj. R^2^ = 0.876, variation explained = 89.2%, GCV = 1.81, Train RMSE = 1.2, Test RMSE = 1.2, AIC = 411, BIC = 469) included the interaction of temperature and R_dark_ (*p* ≤ 0.05), temperature and A_net_ (*p* < 0.001), and the interaction between the NSC concentration and the percentage starch contributes to the total NSC pool (<0.001).

### The relationships of ε_HA_ and ε_OA_ with the carbon metabolism

The apparent autotrophic ^2^H fractionation ε_HA_ was positively related to R_dark_ in 5 of 7 species (not in *P. dioica* and *S. bicolor*, Supplementary Fig. 5) and positively related in all C_3_ but not the C_4_ (*S. bicolor*) species with the percentage R_dark_ contributed to P (i.e., R_dark_ + A_net_R_dark_^−1^; Supplementary Fig. 6). Furthermore, ε_HA_ was positively related to the NSC partitioning into sugar and starch, as expressed in the % that sugar contributes to the total leaf NSC pool (Supplementary Fig. 7), in 5 of the 7 species (not in *O. sativa* and the C_4_ species *S. bicolor*). The observed relationships strongly vary between the different species, with R^2^ ranging, when significant, from 0.37 to 0.86 with R_dark_, from 0.22 to 0.70 with the percentage R_dark_ contributed to P, and from 0.22 to 0.81 with the % that sugar contributes to the total leaf NSC pool (Supplementary Figs. 5–7).

ε_OA_ was negatively related to R_dark_ in 4 of 7 species (not in *Q. pubescens*, *S. hispanica*, and *S. bicolor*, Supplementary Fig. 8) and negatively related in 3 of 7 species (not in *O. sativa*, *Q. pubescens*, *S. hispanica*, and *S. bicolor*) with the percentage R_dark_ contributed to P (Supplementary Fig. 9. Lastly, ε_OA_ was negatively related to the NSC partitioning into sugar and starch (Supplementary Fig. 10) in 3 of the 7 species (*H. vulgare*, *P. dioica*, and *S. cheesmaniae*), and positively related in *Q. pubescens*. As in ε_HA_, the observed relationships strongly vary between the different species, with R^2^ ranging, when significant, from 0.37 to 0.72 with R_dark_, from 0.30 to 0.59 with the percentage R_dark_ contributed to P, and from 0.25 to 0.62 with the % that sugar contributes to the total leaf NSC pool (Supplementary Figs. 8, 9 and 10).

## Discussion

Mortality during heatwaves is strongly driven by drought-induced hydraulic failure (Rowland et al.^55^; Kono et al.^56^). However, even without soil or atmospheric drought, elevated temperatures may lead to significant shifts and perturbations in carbohydrate dynamics, as shown in this study (Fig. 2, Supplementary Tables 3 and 4). As a result, plants may become in the long-term more susceptible to other stressors such as drought, pests and diseases. A_net_ of C_3_ plants decreased at temperatures above 30 °C (Fig. 1, Supplementary Table 1) due to a combination increased respiration, a reduced photosystem II functionality, as indicated by increasing NPQ and decreasing ETR and ΦPSII (Supplementary Fig. 1, Supplementary Table 2), and increasing photorespiration (Keys et al.^57^). The strongly increased respiration (Fig. 1) at high temperatures is needed to support higher metabolic rates (Criddle et al.^25^), thus requiring plants to have more of their carbohydrate pool directly available (Scafaro et al.^58^). This was reflected by a shift towards a higher share of leaf sugars in the total leaf NSC pool with increasing temperature (Fig. 2, Supplementary Tables 3 and 4). Furthermore, these changes in assimilation, respiration, and leaf-level carbohydrate dynamics significantly impacted δ^18^O_LS_, ε_OA,_ δ^2^H_LS_, and ε_HA_.

While some studies found no changes in NSC concentration and composition in response to temperature only at temperatures bellow 20 °C^42–44^ and a recent review comparing NSC temperature response across biomes^45^, we found that high temperatures above 30 °C alone can significantly impact NSC concentration and composition. Therefore, further research should investigate the long-term response of plants to prolonged exposure to temperatures above 30 °C. If plants cannot adjust their respiration rates to chronically high temperatures above a certain, likely species-specific threshold, they might ultimately face carbon starvation if the imbalance between assimilation and respiration rates persists for too long. If the observed increase in leaf sugar δ^2^H, potentially accompanied by a simultaneous decrease in δ^18^O, is translated into the isotopic signature of tree-ring cellulose, this could be used as a new isotope proxy to identify trees with an unfavorable carbon balance, which would be in agreement with recent studies on the subject^11,12^. For instance, this could be used to identify trees that have shifted out of their climatic niche due to climate change. Thus, additional studies to investigate the here observed leaf-level temperature-induced carbohydrate depletion on a whole plant level could contribute to the understanding of the high-temperature response of plants.

The close positive relationship between δ^18^O_LW_ and δ^18^O_LS_ (Fig. 3), explaining explained 64.3% of the observed variation alone in the tested GAM models (Supplementary Table 7), aligns well with the findings of previous studies (Yakir and DeNiro^59^; Roden et al.^4^; Zech et al.^60^). However, we can further demonstrate that the δ^18^O_LS_ and ε_OA_ are temperature-dependent in C_3_ plants (Supplementary Fig. 3), with a smaller ε_OA_ at high temperatures for all except *Q. pubescens* and *S. bicolor* (Supplementary Fig. 3, Supplementary Table 5). A similar temperature response has been demonstrated for cellulose oxygen isotope ratios of aquatic plants (Sternberg and Ellsworth^13^). Our results show that the temperature dependence of ε_OA_ for leaf sugar, while small in comparison to ε_HA,_ must be considered to correctly understand and interpret δ^18^O in plant organic matter, the overall temperature effect on ε_OA_ is −0.11‰ °C^−1^ (Fig. 3).

In contrast, the temperature response of ε_HA_ and δ^2^H_LS_ are non-linear and not driven by changes in leaf water (Fig. 3, Supplementary Fig. 2, Supplementary Table S6), indicating the interplay of different biochemical processes responsible for the apparent photosynthetic ^2^H fractionation. While CO_2_ fixation produces sugar highly depleted in ^2^H (Zhang et al.^61^), higher respiration rates have been found to be related to higher δ^2^H_LS_ (Holloway-Phillips et al.^62^; Lehmann et al.^10^). As the isotopic composition of sucrose derived from transitory starch is the same as that of sucrose synthesized during the day^46^, it is unlikely that sugar:starch partitioning during the daytime is responsible for the observed changes in the leaf sugar isotopic composition. An explanation for this process could be a preferential usage of glyceraldehyde 3-phosphate (GAP) or sugar with ^1^H instead of ^2^H for respiration, similar to the equilibrium tritium isotope effect that has been observed between glucose and hexokinase (Lewis and Schramm^63^), the first enzyme involved in glycolysis. However, other enzymatic reactions, such as glucose-6-phosphate dehydrogenase^47^ or phosphoglucose isomerase^17,48^, among others, could be responsible for the observed respiratory ^2^H enrichment in leaf sugar. The observed ^2^H enrichment in tree-ring cellulose during a reduced or negative carbon balance of trees in response to defoliation or a reduced water access^11,12,49^ supports the findings of this study, where an increase in δ^2^H in carbohydrates is related to a reduced or overall negative C balance. However, also other reactions such as sugar derived from the photorespiratory pathway might influence the observed ^2^H enrichment^50,51^.

**Fig. 5:**
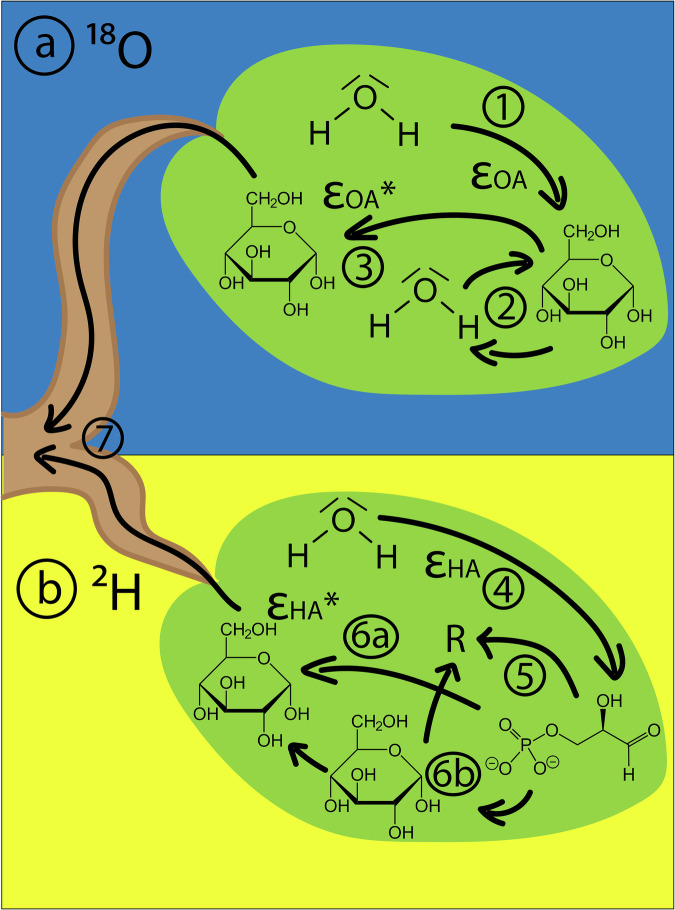
The apparent autotrophic ^2^H and ^18^O fractionation. The leaf-level processes leading to the apparent **a** ^18^O and **b** ^2^H fractionation, ε_OA_* and ε_HA_*, respectively, in leaf sugar.

In Fig. 5, we summarize the suggested main links between a leaf-level carbon dynamic with the ^18^O and ^2^H fractionation: (1) The initial photosynthetic ^18^O fractionation ε_OA_ from H_2_O to leaf sugar leads to an ^18^O-enriched leaf sugar, without species-specific differences. This photosynthetic ^18^O enrichment is likely reduced under elevated temperatures. (2) Increased isotopic exchange reactions between leaf sugar and leaf water under elevated metabolic rates further alter the initial leaf sugar δ^18^O, leading to leaf sugar less ^18^O enriched and therefore (3) alter the apparent autotrophic ^18^O fractionation, ε_OA_*. (4) The strong photosynthetic ^2^H fractionation ε_HA_ from H_2_O to leaf sugar leads to a strong ^2^H depletion compared to the leaf water, with a large amplitude between species, which must be already present in glyceraldehyde 3-phosphate (G3P). During daytime respiration (R), (5) isotopically lighter G3P might be preferably used as a substrate, (6a) leading to a ^2^H enriched leaf sugar which is synthetized from the remaining ^2^H enriched G3P pool, leading therefore an altered apparent autotrophic ^2^H fractionation, ε_HA_*. However, (6b) glycolysis might further contribute to a further ^2^H enrichment in the remaining sugar pool, especially in times of high respiratory demand. (7) This “processed” leaf sugar is eventually exported to the long-term sinks, where its isotopic signature, after being further altered during the transport and in the sink tissues by various processes, influences the δ^18^O and δ^2^H of the tree-ring cellulose.

In conclusion, we can demonstrate that air temperatures above 30 °C substantially alter the leaf-level carbon (i.e., A_net_ and R_dark_) and carbohydrate dynamics C_3_ plants. This was associated with a reduction in the total NSC pool size and a higher proportion of the carbohydrates being stored as directly metabolically available sugars and less as starch reserves. This was reflected in increased δ^2^H and a decreased δ^18^O in leaf sugar. The C_4_ plant maintained low respiration and high assimilation rates even at temperatures above 35 °C, thus, no temperature response of leaf sugar δ^2^H was observed. Finally, by using the collected data on gas exchange and NSC concentrationin generalized additive models, we were able to precisely reconstruct the measured O and H isotopic composition of the leaf sugar, and therefore confirm the close relationship between plant carbohydrate metabolism and stable isotope fractionation beyond source water effects, especially for δ^2^H in leaf sugar. We speculate that the increased respiration rate likely caused a ^2^H enrichment in the remaining sugar pool, leading to a reduced apparent autotrophic ^2^H fractionation (ε_HA_) from leaf water to leaf sugar when temperatures increased, with leaf water having no impact on ε_HA_. Furthermore, since the partitioning between sugar and starch changed in *S. bicolor* with rising temperatures but ε_HA_ did not, while the partitioning in *O. sativa* did not change but ε_HA_ did, we assume that the partitioning process is likely not involved in the observed changes in ε_HA_. This is supported by the finding that the δ^2^H of sugar deriving from transitory starch during the nighttime does not differ from the δ^2^H of sugar derifing from G3P during the daytime^46^. However, the metabolic changes for the observed respiratory ^2^H enrichment of the leaf sugar, at least in some species, coincide with changes in the NSC partitioning. On the other hand, the observed relative depletion of leaf sugar δ^18^O with increasing temperatures was caused by a combination of a decreasing leaf water δ^18^O and a reduced apparent ^18^O fractionation (ε_OA_) from leaf water to leaf sugar. Therefore, increasing oxygen isotopic exchange reactions between leaf water and leaf sugar are likely the main driver behind leaf sugar δ^18^O.

Position-specific δ^2^H analysis of sucrose^17,47^ and other metabolites will be needed to identify the exact biochemical reactions for the observed respiratory ^2^H enrichment and ^18^O depletion with increasing temperatures in leaf sugar, and how they are later transferred into the tree-ring archive.

## Methods

### Experimental design and plant growing conditions

To isolate the effects of rising temperature under a constant VPD on leaf physiology, metabolism, and the corresponding triple isotope fractionation, we established a specific experimental and sampling design. We selected six C_3_ and one C_4_ plant species, with different biochemical and anatomical features as well as temperature adaptations. For the C_3_ species, we selected two trees, *Quercus pubescens* WILLD. and *Phytolacca dioica* L.; two grasses, *Hordeum vulgare* L. and *Oryza sativa* L.; and two forbs, *Salvia hispanica* L. and *Solanum cheesmaniae* (RILEY) FOSBURG. For the C_4_ plant, we selected the grass *Sorghum bicolor* (L.) MOENCH. With this species selection, we aimed to make the results of this study relevant to a broad field of plant sciences, including plant ecophysiology, forestry and forest ecology, as well as agriculture. Starting in November 2021, we grew replicates of plants (*n* = 3 for *Quercus pubescens*, *Phytolacca dioica*, *Solanum cheesmaniae*, *Sorghum bicolor*, *n* = 50 for *Hordeum vulgare* and *Oryza sativa*) for 2 to 3 months in a climate chamber (Plant Growth Chamber PGR15, Controlled Environments Limited (CONVIRON), Winnipeg, Manitoba, Canada) at the Swiss Federal Institute for Forest, Snow, and Landscape Research WSL, at a temperature of 25 °C, a VPD of 1 kPa, and with a photosynthetic active radiation (PAR) of 800 µmol of photons m^−2^ s^−1^ for 18 h a day, followed by 6 h of nighttime in complete darkness at a temperature of 20 °C. The low VPD was chosen to avoid plant stress due to VPD while changing temperature and to ensure saturated intercellular airspaces in the leaves during gas exchange measurements (Diao et al.^64^). After the initial growth period, the actual treatment period of seven weeks started. To reduce the pool of old leaf NSC between each of the seven temperature cycles, plants were kept in the dark at 20 °C for 48 h, similar to (Wieloch et al.^17^). This depletion of old NSC was used to obtain an unadulterated stable isotope (^2^H, ^18^O) signal, reflecting the plant physiological conditions at the respective temperature, and thus avoiding autocorrelation. During the start of each week, we exposed the plants for 5 days to 18 h of a constant daytime temperature, starting at 10 °C and subsequently increasing to 40 °C in 5 °C steps every week (Fig. 1a). This allowed the plants to acclimate to each of the studied temperatures. The nighttime temperature for the daytime 10, 15, and 20 °C treatments was the same as the daytime temperature and a VPD of 1 kPa to avoid chilling damage. Nighttime temperatures for 25, 30, 35, and 40 °C were all set to 20 °C and a VPD of 1 kPa to enable the plants to recover their photosynthetic machinery overnight. After four days of treatment, we sampled leaves for non-structural carbohydrates (NSC) and stable isotope analysis in the early afternoon. After 5 days, we conducted gas exchange and fluorescence measurements. The separation of leaf sampling and gas exchange measurements was done to avoid any influence of introduced unstable conditions during gas exchange measurements. At 40 °C, one of the three replicates of *S. cheesmaniae* and about two-thirds of the *H. vulgare* plants died.

### Plant physiological measurements

After 5 days of exposure to each temperature, one leaf per plant was dark adapted by gently folding aluminum foil around it for at least 20 min. After that, dark respiration (R_dark_) and dark-adapted fluorescence were measured using a Li-6800 (LI-COR Biosciences, Lincoln, NE, USA) at the same conditions (CO_2_, RH, and temperature) as present in the climate chamber but without light. After that, photosynthetic active radiation (PAR) of the LI-6800 was set to the value of the climate chamber (CO_2_, RH, and temperature still the same as in the climate chamber), and a light-adapted leaf of the same plant in close proximity was fixed into the measuring chamber. The leaf and the chamber were allowed to equilibrate for 15 to 20 min until gas exchange reached steady state before measuring A_net_, stomatal conductance (g_s_), as well as light-adapted fluorescence. Gross photosynthesis (P) was calculated as the sum of A_net_ and R_dark_. With the dark- and light-adapted fluorescence measurements, the LI-6800 automatically calculated the non-photochemical quenching (NPQ), the photosynthetic efficiency of photosystem II (Fv/Fm), the quantum yield of photosystem II (ΦPSII), and the electron transport rate of photosystem II (ETR). All measured values can be found in Supplementary Tables 1 and 2.

### Sampling of leaf material

For each temperature step, three samples each consist of several light-exposed leaves were collected from each plant in the early afternoon using scissors. Leaf material was sampled in excess to make sure there was enough plant material and water (>2 mL of water for all samples) to avoid methodological bias during water extraction (Diao et al.^65^). The fully developed leaves were immediately transferred into individual gas-tight 12 ml glass vials (Prod. No. 738W, Exetainer, Labco, Lampeter, UK, stored on dry ice, and transferred in a −20 °C freezer until leaf water extraction.

### Extraction of leaf water and sugars

Leaf water was cryogenically extracted using a hot water bath at 80 °C and liquid nitrogen following established protocols (West et al.^52^; Diao et al.^65^), and a schematic overview of the extraction unit can be found in Diao et al.^65^. In short, glass vials with the frozen leaf samples, which were blocked by PP fiber filters (Nozzle protection filter, Socorex Isba SA, Ecublens, Switzerland), were attached to the extraction unit. The sample vials were submerged in a water bath at 80 °C, while u-shaped glass sample collection tubes were submerged in liquid nitrogen to instantly trap arriving water vapor. The extraction procedure was conducted under vacuum (<0.02 mbar) for 2 h^52,53^. Afterwards, when the extraction was complete, the extraction line was filled with dry nitrogen gas to ambient pressure. The u-shaped glass sample collection tubes were then taken out of the extraction line and closed with rubber plugs. The thawed water samples were transferred to 2 mL glass vials (Infochroma AG, Goldau, Switzerland) with pipettes and stored in glass vials at −20 °C until isotope analysis.

After the water extraction, the dried leaf material was ground (MM400, Retsch, Haan, Germany), and the bulk leaf sugar fraction for isotope analysis was then extracted from 100 mg of leaf powder following established protocols (Rinne et al.^66^; Lehmann et al.^67^). First, the ground leaf material was mixed with deionized water in a 2 ml reaction vial and the water-soluble content was extracted at 85 °C for 30 min. Leaf sugars were then purified from the water-soluble content using ion exchange cartridges (OnGuard II A, H and P, Dionex, Thermo Fisher Scientific, Bremen, Germany). Finally, leaf sugar material was acquired by freeze-drying the purified sugar solutions.

### δ^2^H and δ^18^O analyses of leaf water

The δ^2^H and δ^18^O of water (δ^2^H_LW_ and δ^18^O_LW_) samples were measured with a high-temperature conversion elemental analyzer coupled to a DeltaPlus XP isotope ratio mass spectrometer (TC/EA-IRMS; Finnigan MAT, Bremen, Germany), and the isotope ratios are reported in per mille (‰) relative to Vienna Standard Mean Ocean Water (VSMOW). Calibration was done using a range of labortory standard waters with different δ^2^H and δ^18^O values (B2193, δ^2^H = −98.32‰, δ^18^O = −12.34‰; and Fiji Artesian Water, δ^2^H = −42.85‰, δ^18^O = −6.41‰), respectively, resulting in a precision of 2‰ for δ^2^H and 0.3‰ for δ^18^O. All the obtained values can be found in Supplementary Table 5.

### δ^2^H and δ^18^O analyses of sugars using a hot water vapor equilibration method

The here used procedure originates mainly from the description in Schuler et al.^8^. δ^2^H of sugars were analyzed according to the previously developed hot water vapor equilibration method (Schuler et al.^68^). Subsequently, as described by Schuler et al.^8^, dry sugar samples were dissolved in water, with a target concentration of 1 mg sugar per 20 µL water. Two identical sets of each sugar sample, with 1 mg sample material each, were prepared by pipetting 20 µL sugar solution into pre-weighed 5 × 9 mm silver foil capsules (Prod. No. SA76981106, Säntis, Switzerland). Sugar samples for δ^18^O measurements were prepared by transferring 20 µL sugar solution of the same solution into pre-weighed 3.3 × 5 mm silver foil capsules (Prod. No. SA76980506, Säntis). All samples were then frozen at −20 °C, freeze-dried with a condenser temperature of −50 °C, and the duplicates for δ^2^H measurements were packed into a second 5 × 9 mm silver foil capsule. Sugar samples were stored in a desiccator at low relative humidity (2–5%) until δ^2^H and δ^18^O measurements.

For the δ^2^H measurements, the sets of duplicates were then equilibrated with hot water vapor by evaporating two isotopically distinct waters (δ^2^H water 1 = −160‰ and δ^2^H water 2 = −428‰) at 130 °C (Schuler et al.^68^). After 2 h, the samples were dried with dry nitrogen gas (N25.0, Prod. No. 2220912, PanGas AG, Dagmersellen, Switzerland) for 2 h at 130 °C. After that, they were immediately transferred into a Zero Blank Autosampler (N.C. Technologies S.r.l., Milano, Italy), which was installed on a sample port of a high-temperature elemental analyzer system. The latter was coupled via a ConFlo III referencing interface to a Delta^Plus^ XP IRMS (TC/EA-IRMS, Finnigan MAT, Bremen, Germany). The autosampler was evacuated to 0.01 mbar and filled with dry helium gas. The samples were pyrolysed in a reactor according to Gehre et al.^69^, and carried in a flow of dry helium (150 ml min^−1^) to the IRMS. Raw δ^2^H values were offset corrected using polyethylene foil standards (IAEA-CH-7 polyethylene foil, International Atomic Energy Agency, Vienna, Austria; SD < 0.7‰ within one run). δ^18^O measurements were done according to established protocols (Weigt et al.^70^; Lehmann et al.^67^) with a PYRO cube (Elementar, Hanau, Germany, precision ~0.3‰).

### Calculation of the non-exchangeable hydrogen isotope ratio (δ^2^H_ne_), ε_OA,_ ε_HA_

The here used procedure originates mainly from the description in Schuler et al.^8^. All isotope ratios (δ) were calculated as given in Eq. 1 (Coplen^71^):

1 $${\rm{\delta }}=\,\frac{{{\rm{R}}}_{{\rm{Sample}}}-{{\rm{R}}}_{{\rm{Standard}}}}{{{\rm{R}}}_{{\rm{Standard}}}}$$

where R = ^2^H/^1^H of the sample (R_Sample_) and of Vienna Standard Mean Ocean Water (VSMOW2; R_Standard_) as the standard defining the international isotope scale. To express the resulting δ in permil (‰), results were multiplied by 1000.

According to Filot et al.^72^, the %-proportion of exchanged hydrogen during the equilibrations (x_e_, Eq. 2) can be calculated as:

2 $${{\rm{x}}}_{{\rm{e}}}=\,\frac{{{\rm{\delta }}}^{2}{{\rm{H}}}_{{\rm{e}}1}-\,{{\rm{\delta }}}^{2}{{\rm{H}}}_{{\rm{e}}2}\,}{{{\rm{\alpha }}}_{{\rm{e}}-{\rm{w}}}\,\cdot \,({{\rm{\delta }}}^{2}{{\rm{H}}}_{{\rm{w}}1}-\,{{\rm{\delta }}}^{2}{{\rm{H}}}_{{\rm{w}}2})}\,$$

where δ^2^H_e1_ and δ^2^H_e2_ are the measured δ^2^H values of the two equilibrated subsamples, δ^2^H_w1_ and δ^2^H_w2_ are the δ^2^H values of the two waters used, and α_e-w_ is the fractionation factor of 1.082, which is the same for sugars and cellulose (Filot et al.^72^; Schuler et al.^68^). Typical x_e_ values for pure sugars are between 0.32 and 0.36 (Schuler et al.^68^).

δ^2^H_ne_ can then be calculated with Eq. 3 using one of the two equilibrations (equilibration one in this example, δ^2^H_e1_ and δ^2^H_w1_):

3 $${{\rm{\delta }}}^{2}{{\rm{H}}}_{\mathrm{ne}}=\frac{{{\rm{\delta }}}^{2}{{\rm{H}}}_{{\rm{e}}1}\,-\,{{\rm{x}}}_{{\rm{e}}}\,\cdot \,{{\rm{\alpha }}}_{{\rm{e}}-{\rm{w}}}\,\cdot \,{{\rm{\delta }}}^{2}{{\rm{H}}}_{{\rm{w}}1\,}-\,1000\,\cdot \,{{\rm{x}}}_{{\rm{e}}}\,\cdot \,({{\rm{\alpha }}}_{{\rm{e}}-{\rm{w}}}\,-\,1)\,}{1-{{\rm{x}}}_{{\rm{e}}}}$$

Three sucrose samples for the equilibrations of leaf sugars and three cellulose samples for the equilibrations of the twig xylem cellulose, each measured in triplicates, were used as internal reference material to calibrate the results. For the sake of simplicity, δ^2^H has been used throughout the manuscript instead of δ^2^H_ne_.

The apparent autotrophic fractionation factors between precursor and product (^18^O = ε_OA_, and ^2^H = ε_HA_) were calculated with Eq. 4 and Eq. 5, respectively:

4 $${{\rm{\varepsilon }}}_{{\rm{OA}}}={{\rm{\delta }}}^{18}{{\rm{O}}}_{{\rm{leaf\; sugar}}}-{{\rm{\delta }}}^{18}{{\rm{O}}}_{{\rm{leaf\; water}}}$$

5 $${{\rm{\varepsilon }}}_{{\rm{HA}}}={{\rm{\delta }}}^{2}{{\rm{H}}}_{{\rm{leaf\; sugar}}}-{{\rm{\delta }}}^{2}{{\rm{H}}}_{{\rm{leaf\; water}}}$$

As in Schuler et al.^8^, the two biological fractionation factors ε_HA_ and ε_OA_ were expressed as the actual difference between the δ^18^O, and δ^2^H of leaf sugars and the δ^18^O and δ^2^H of leaf water, respectively. All the obtained values can be found in Supplementary Table 5.

### Leaf-level non-structural carbohydrates (NSC) analysis

The leaf material for NSC analysis derives from the material which was dried during the cryogenic water extraction at 80 °C. Then, leaves were ground in fine powder and NSC concentrations were measured following previously established protocols (Hoch et al.^73^; Schönbeck et al.^74^). Ten to twelve mg of finely ground leaf material were heated in 2 mL distilled water for 30 min. An aliquot of 200 µL was treated with invertase from baker’s yeast (S. cerevisiae, Sigma-Aldrich Chemie GmbH, Germany) for an hour to degrade sucrose and convert fructose into glucose. The sugar concentration was determined at 340 nm in a 96-well plate spectrophotometer (Thermo Fisher Scientific Multiskan GO, Finland) after an enzymatic conversion to gluconate-6-phosphate of about 35 min, using glucose Assay Reagent (Sigma-Aldrich Chemie GmbH, Germany) and Isomerase from baker’s yeast (S. cerevisiae, Sigma-Aldrich Chemie GmbH, Germany). The total amount of NSC was measured by taking an aliquot of 500 µL of the extract (including starch and sugar) and treated for 15 h at 49 °C with Amyloglucosidase from Aspergillus niger (Sigma-Aldrich Chemie GmbH, Germany) to digest starch into glucose. Total glucose (corresponding to total NSC concentration) was determined using a spectrophotometer, as explained above. The starch concentration was calculated as the total NSC subtracted by the sugar concentration. Standard solutions, including pure starch, glucose, fructose, sucrose, and standard plant powder (Orchard leaves, Leco, USA), were used as references for the comparison and reproducibility of the results between runs. All the obtained values can be found in Supplementary Table 3.

### Statistical analyses

Statistical analyses were performed using R version 4.1.2 (R.Core.Team^75^). Linear and polynomial models, Mann-Whitney-U-Test, and Welch’s t-test implemented using *basic R* and *ggplot2* (Wickham^76^) were used to determine the leaf-level physiological and NSC temperature response. The final assembly of the graphs was done using the R package *patchwork* (Pedersen^77^). Least Absolute Shrinkage and Selection Operator (Lasso) regressions to identify important variables were performed using the R package *glmnet* (Tay et al.^78^). Subsequently, to analyze the relationship between isotope fractionation and leaf physiological variables, we developed generalized additive models (GAMs) using the R package *mgcv* (Wood^79^), which allowed us to capture any non-linear relationships and thus identify the main drivers behind hydrogen and oxygen isotope fractionation. In total, we tested 34 different models with increasing complexity for δ^2^H and 19 for δ^18^O in leaf sugar. The models included smooth terms for the variables of interest, and interactions were modeled using tensor product smooths and were trained on 80% and tested on 20% of the dataset. Whether the simulated leaf sugar isotope temperature response differed significantly from the measured leaf sugar isotope temperature response by testing the significance of the interaction term in an ANOVA. Model visualization and diagnostics were performed using ggplot2, ggplotify, and cowplot to combine plots. Lastly, we tested the relationships between ε_HA_ and ε_OA_, respectively, with R_dark_, the % R_dark_ contributes to P (R_dark_ + A_net_), as well as the partitioning between sugar and starch using Pearson’s correlation coefficients (cor.test from *dplyr*^54^) within each species.

## Supplementary information

Supplementary Information

## Data Availability

All data used for the analysis are available in the tables of the supporting information, and can further be downloaded from www.envidat.ch after the publication of the manuscript.
