# Supplementary material for: Hot and Hungry - High temperatures induce changes in leaf carbon dynamics and sugar isotope fingerprints
Source: NPJ Sci Plants. 2025 Dec 1;1(1):12. doi: 10.1038/s44383-025-00012-6 (PMC12669037; doi:10.1038/s44383-025-00012-6)
Supplement: Supplementary file 1 — Supplementary Information [file 44383_2025_12_MOESM1_ESM.pdf]

Hot and Hungry: High temperatures induce changes in leaf carbon dynamics and sugar isotope fingerprints

Philipp Schuler[1,2,3], Margaux Didion-Gency[4], Valentina Vitali[1,5], Matthias Saurer[1], Manuela Oettli[1], Haoyu Diao[1], Nina Buchmann[2], Arthur Gessler[1,2], Marco M. Lehmann[1]

[1]Forest and Soil EcologySwiss Federal Institute for Forest, Snow and Landscape Research WSL, 8903 Birmensdorf, Switzerland

[2]Department of Environmental Systems Science, ETH Zurich, 8006 Zurich, Switzerland

[3]Plant Ecology Research Laboratory PERL, School of Architecture, Civil and Environmental Engineering ENAC, EPFL, 1015 Lausanne, Switzerland

[4]Ecological and Forestry Applications Research Center (CREAF), E-08193 Cerdanyola del Valley, Spain

[5] Forest Ecology, Institute of Terrestrial Ecosystems, Department of Environmental Systems Science, ETH Zürich 8092 Zürich Switzerland

Philipp Schuler https://orcid.org/0000-0002-5711-2535

Margaux Didion-Gency https://orcid.org/0000-0001-8967-3655

Valentina Vitali https://orcid.org/0000-0002-3045-6178

Matthias Saurer https://orcid.org/0000-0002-3954-3534

Haoyu Diao https://orcid.org/0000-0002-8404-1998

Arthur Gessler https://orcid.org/0000-0002-1910-9589

Nina Buchmann https://orcid.org/0000-0003-0826-2980

Marco M. Lehmann https://orcid.org/0000-0003-2962-3351

Supplementary Information

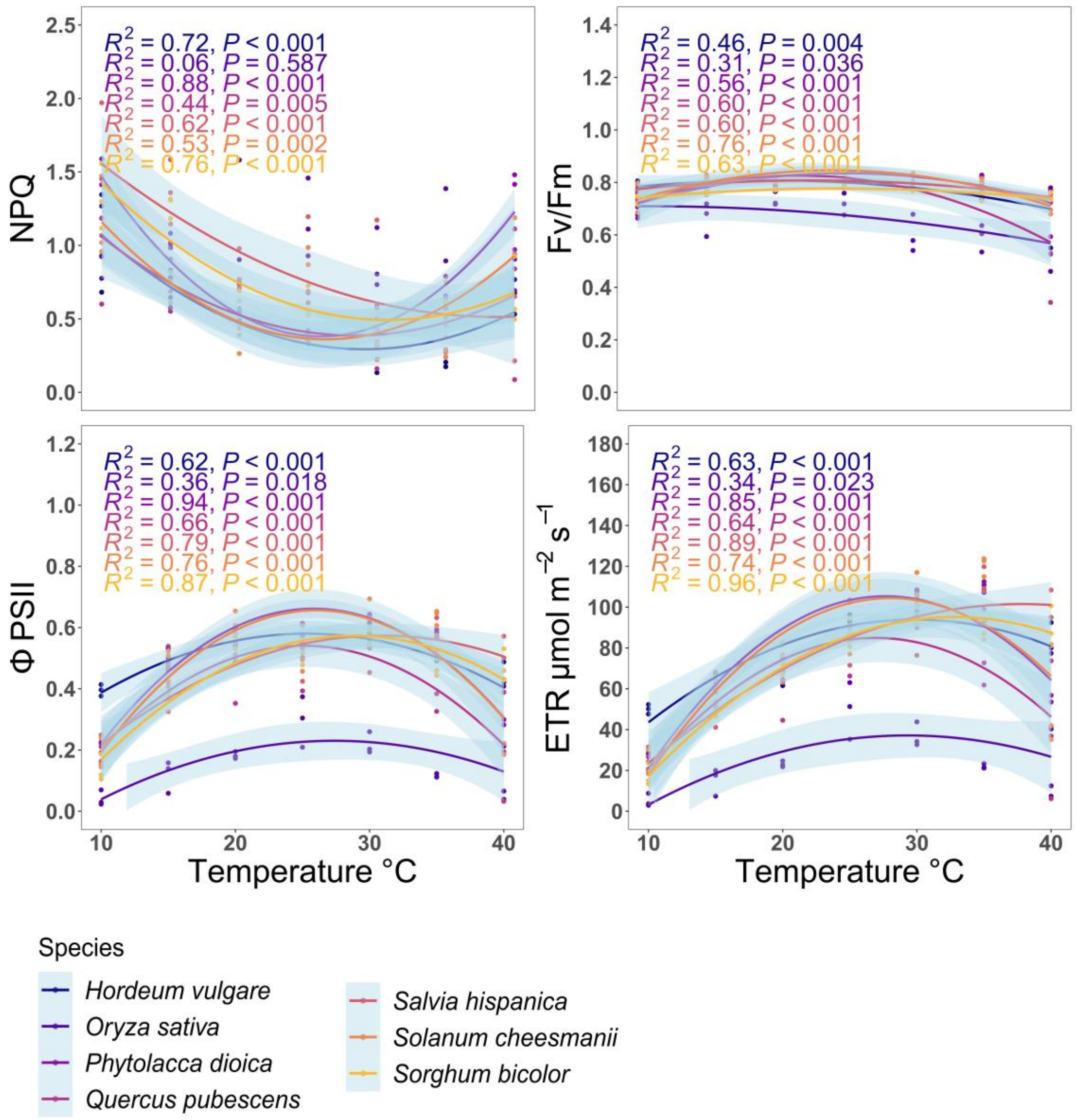

Supplementary Figure 1: Temperature response of non-photochemical quenching (NPQ), the maximum quantum efficiency of photosystem II (Fv/Fm), the quantum yield of PSII (ΦPSII), and the electron transport rate (ETR). Species are indicated by colours, quadratic model depicting the relationship are shown only for species showing a significant response ($p \leq 0.05$), and the light blue shading denotes the 95% confidence level interval for predictions of the quadratic fit.

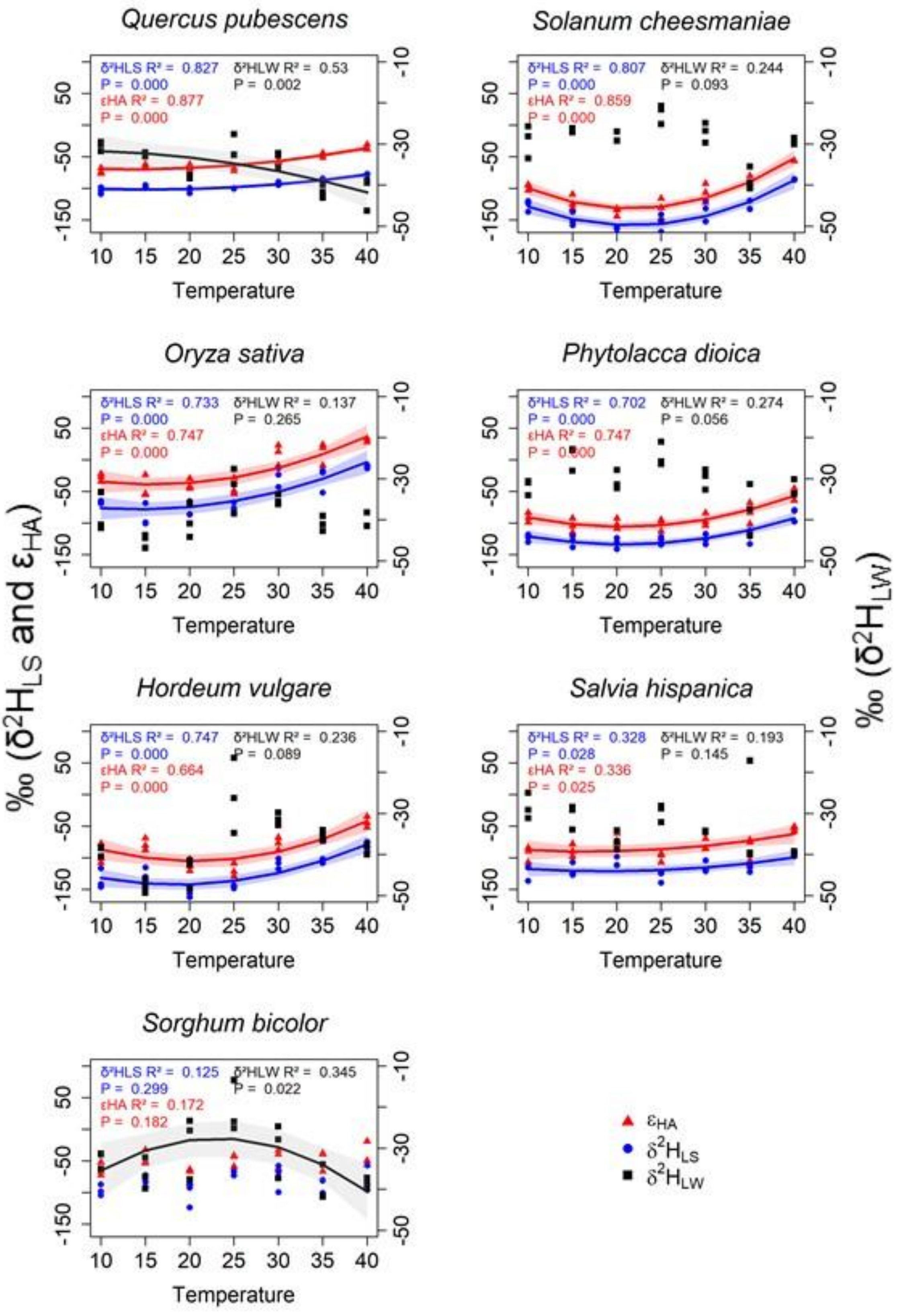

Supplementary Figure 2: Species-specific temperature response of the apparent autotrophic ^{2}H fractionation factor ε_{HA}, the hydrogen isotope value of the leaf sugar

δ^2H_{LS}, and the hydrogen isotope value of the leaf water δ^2H_{LW}. Each species' isotopic response to temperature was fitted using quadratic regressions, with R^2 values and significance (p-values) reported for each variable. Only significant temperature responses ($p < 0.05$) are shown with fitted lines and 95% confidence intervals. The goodness of fit, represented by R^2 values, ranges from moderate to high, indicating that the quadratic models explain a substantial proportion of the variability in the data. Non-significant models ($p > 0.05$) are omitted to emphasize only robust trends.

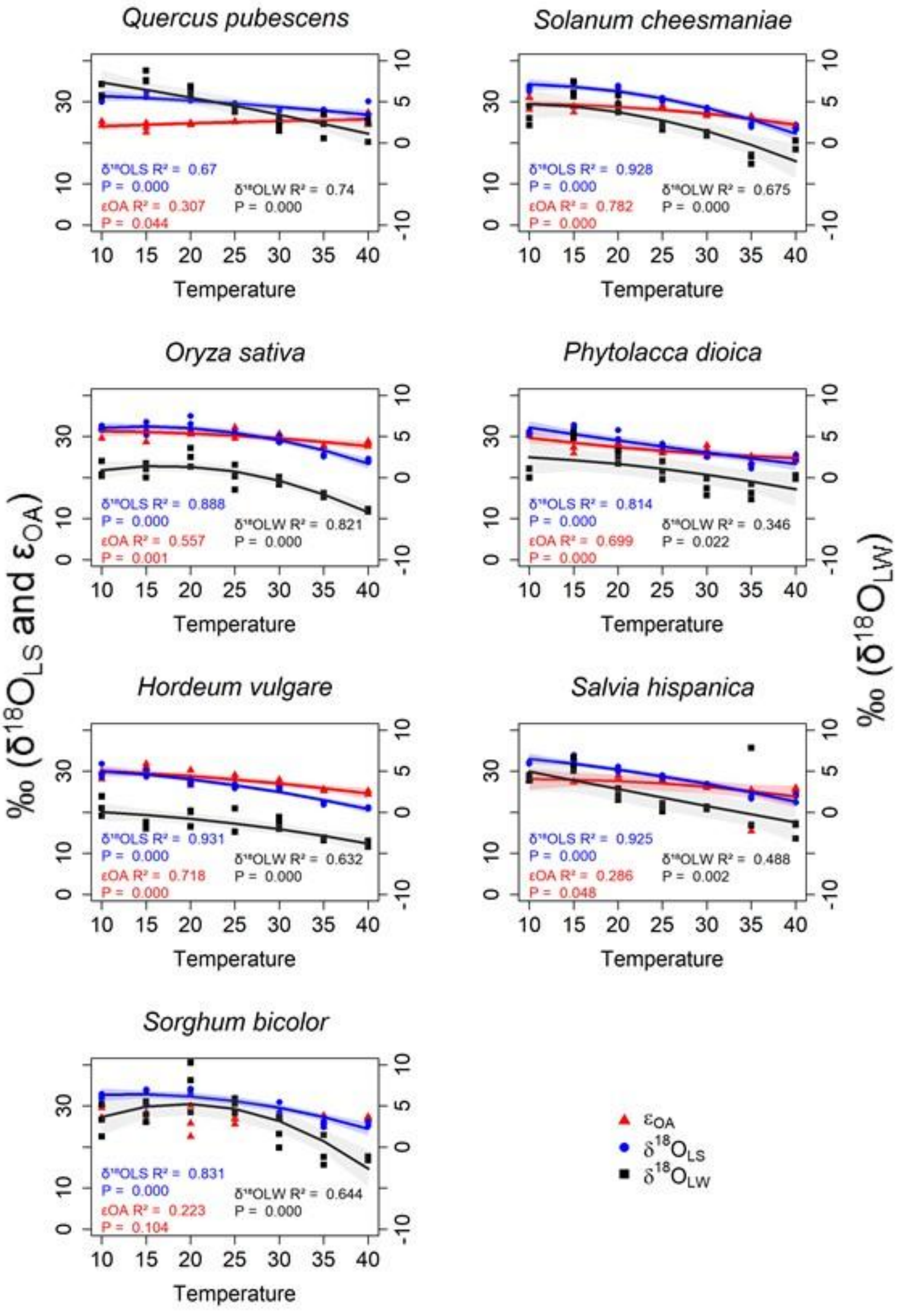

Supplementary Figure 3: Species-specific temperature response of the apparent autotrophic ^{18}O fractionation factor ε_{OA}, the hydrogen isotope value of the leaf sugar $\delta^{18}O_{LS}$, and the hydrogen isotope value of the leaf water $\delta^{18}O_{LW}$. Each species' isotopic response to temperature was fitted using quadratic regressions, with R^2 values and

significance (p-values) reported for each variable. Only significant temperature responses ($p < 0.05$) are shown with fitted lines and 95% confidence intervals. The goodness of fit, represented by R^2 values, ranges from moderate to high, indicating that the quadratic models explain a substantial proportion of the variability in the data. Non-significant models ($p > 0.05$) are omitted to emphasize only robust trends.

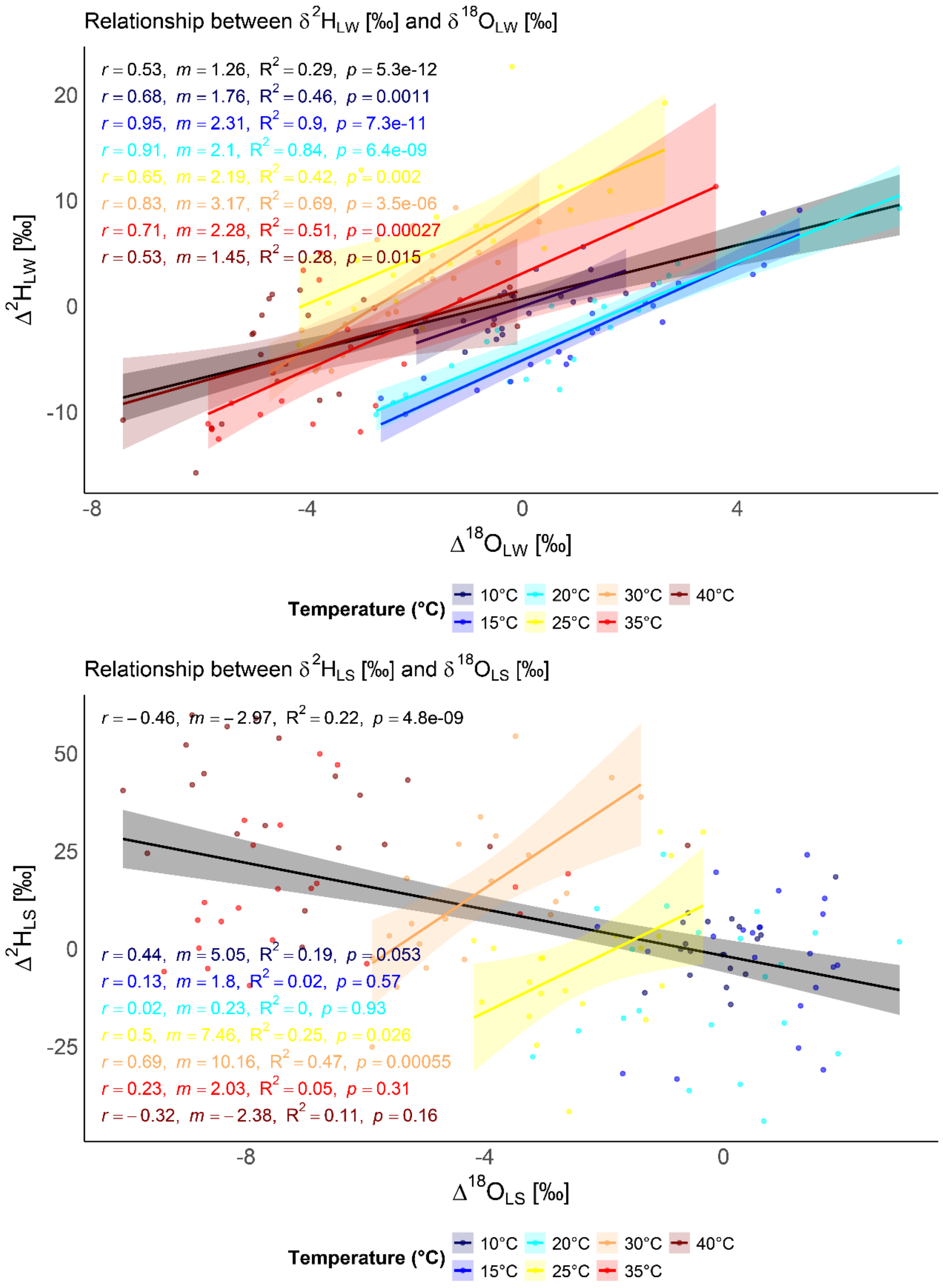

Supplementary Figure 4: Species-specific (colors from blue to red) and overall (black) covariation between Δ^2H and $\Delta^{18}O$ (normalized to the average species-specific value at 10°C) in leaf water (upper panel) and leaf sugar (lower panel). Only significant relationships are displayed with linear regressions. The shaded area represents the 95% confidence interval.

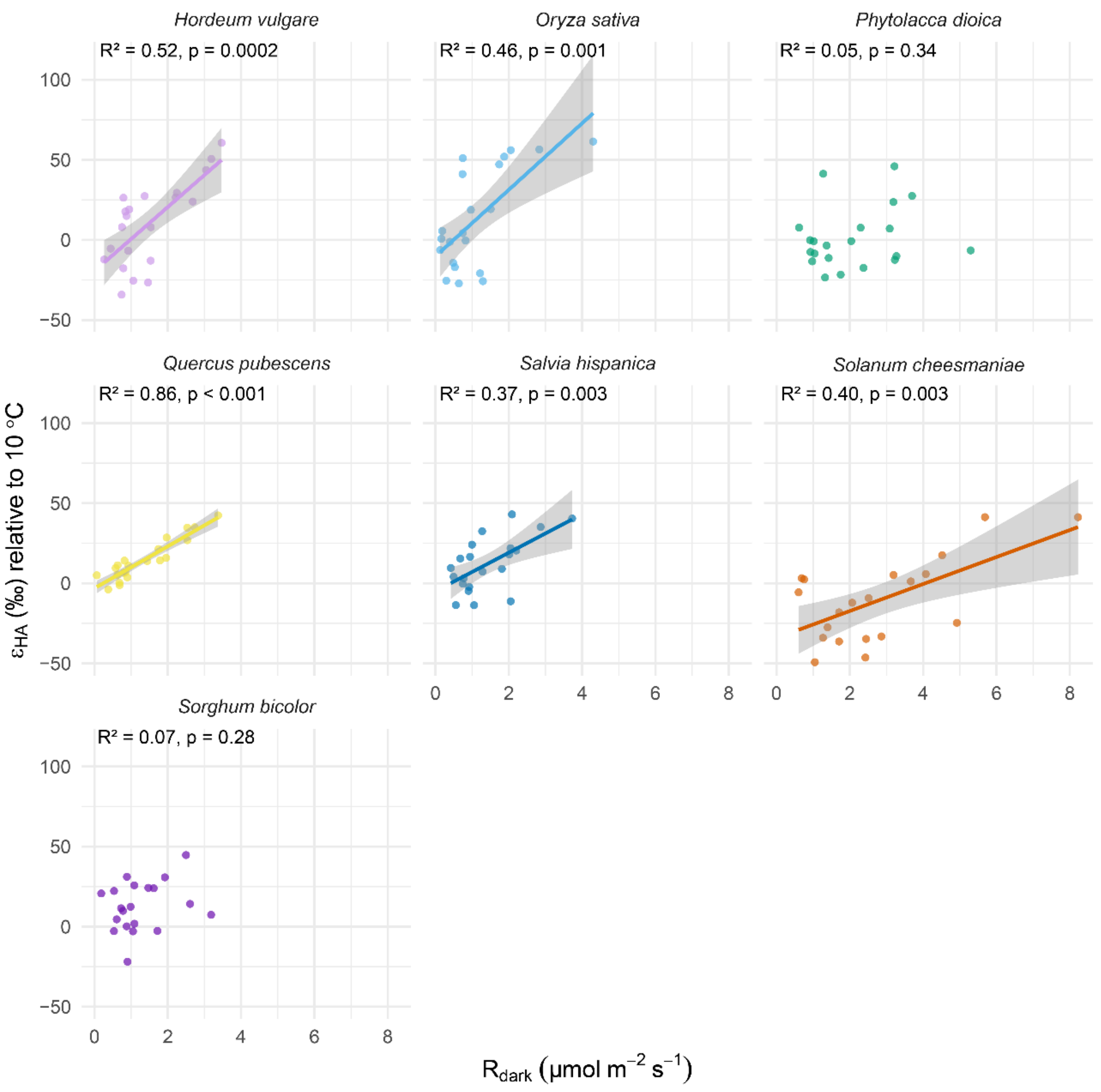

Supplementary Figure 5: Species-specific relative response ε_{HA} to R_{dark}. Only significant relationships are displayed with linear regressions. The shaded area represents the 95% confidence interval.

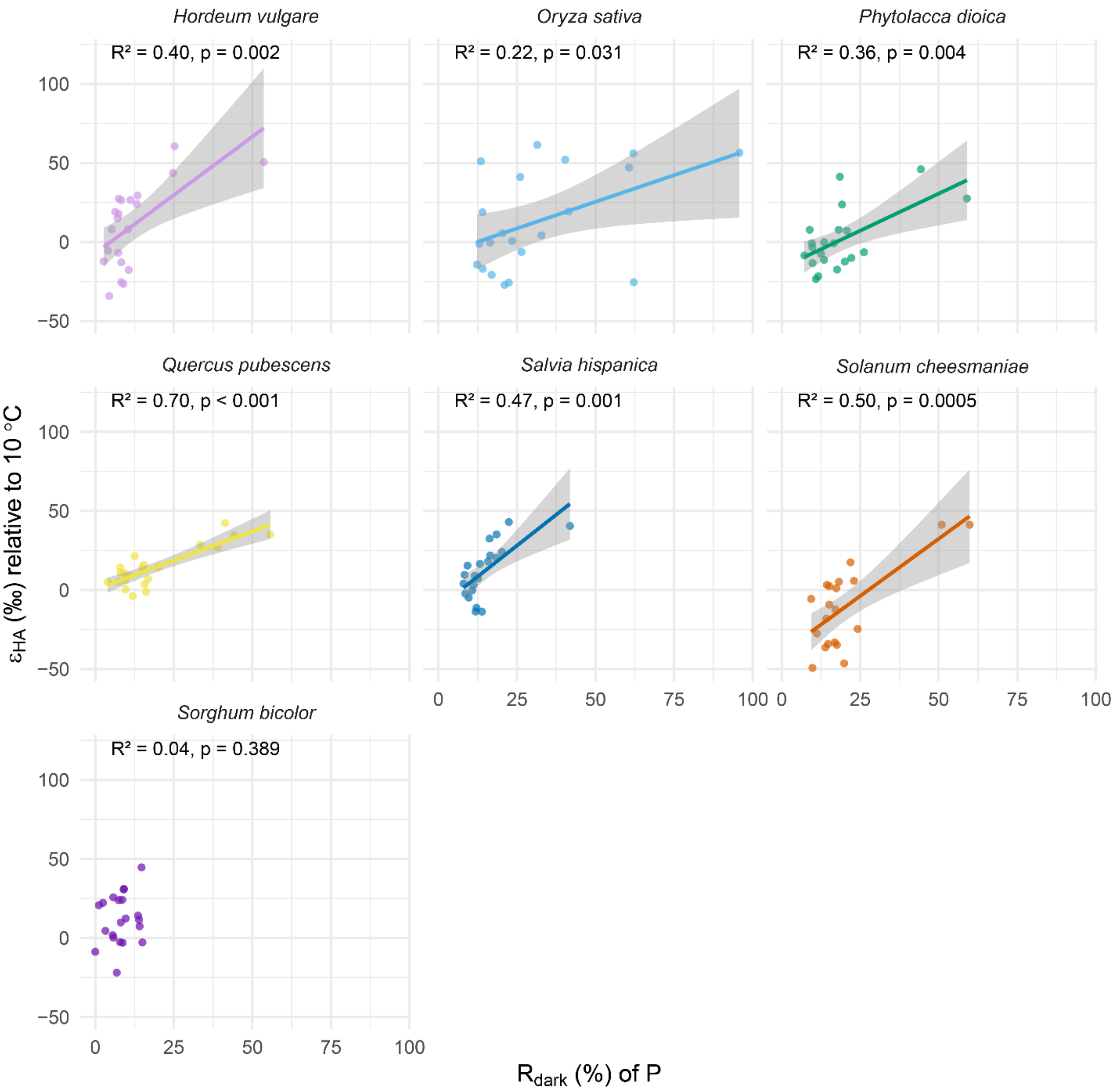

Supplementary Figure 6: Species-specific relative response ε_{HA} to R_{dark} in % of total photosynthesis (P). Only significant relationships are displayed with linear regressions. The shaded area represents the 95% confidence interval.

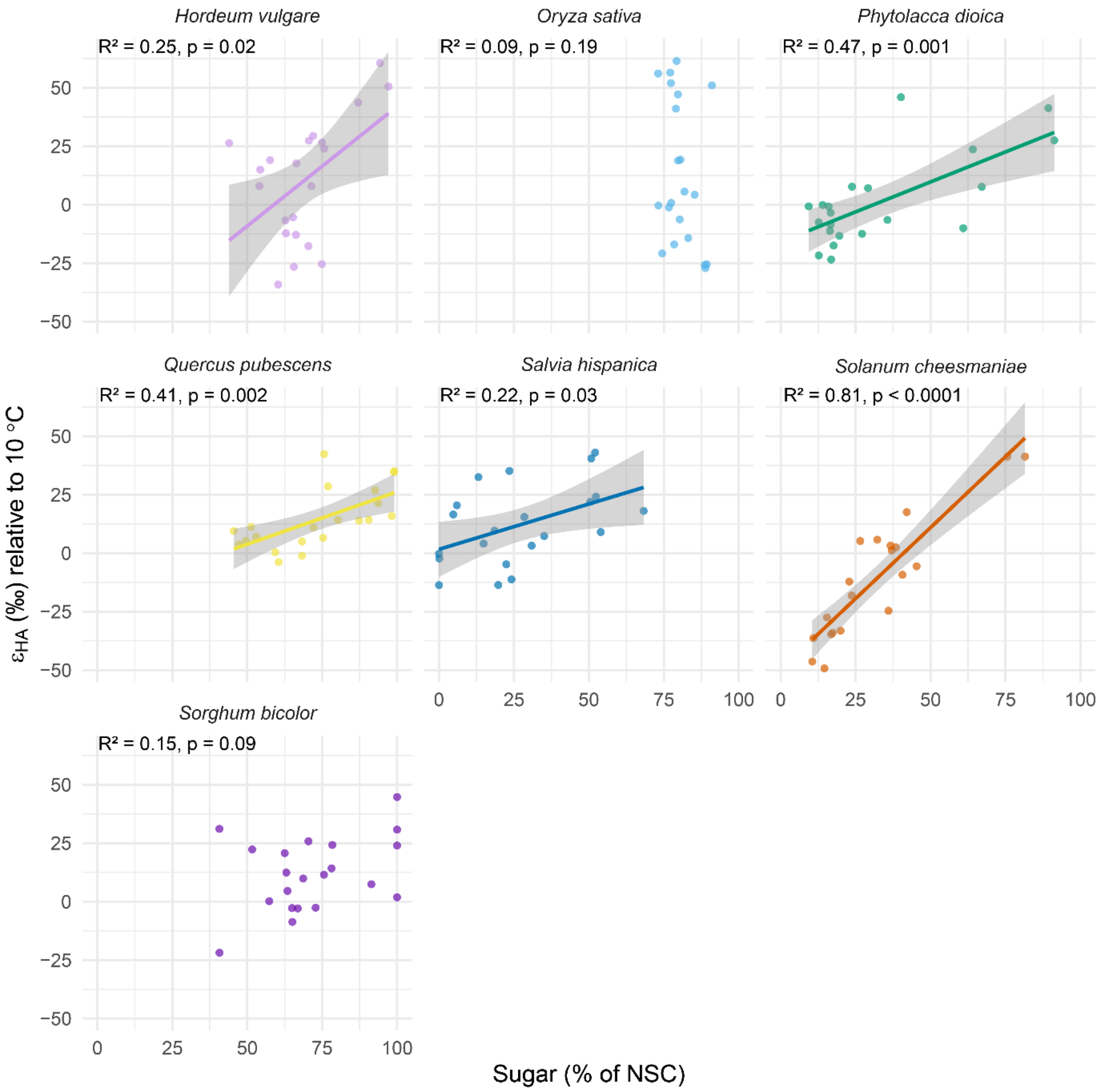

Supplementary Figure 7: Species-specific relative response ε_{HA} to the percentage sugar contributes to the whole leaf NSC pool. Only significant relationships are displayed with linear regressions. The shaded area represents the 95% confidence interval.

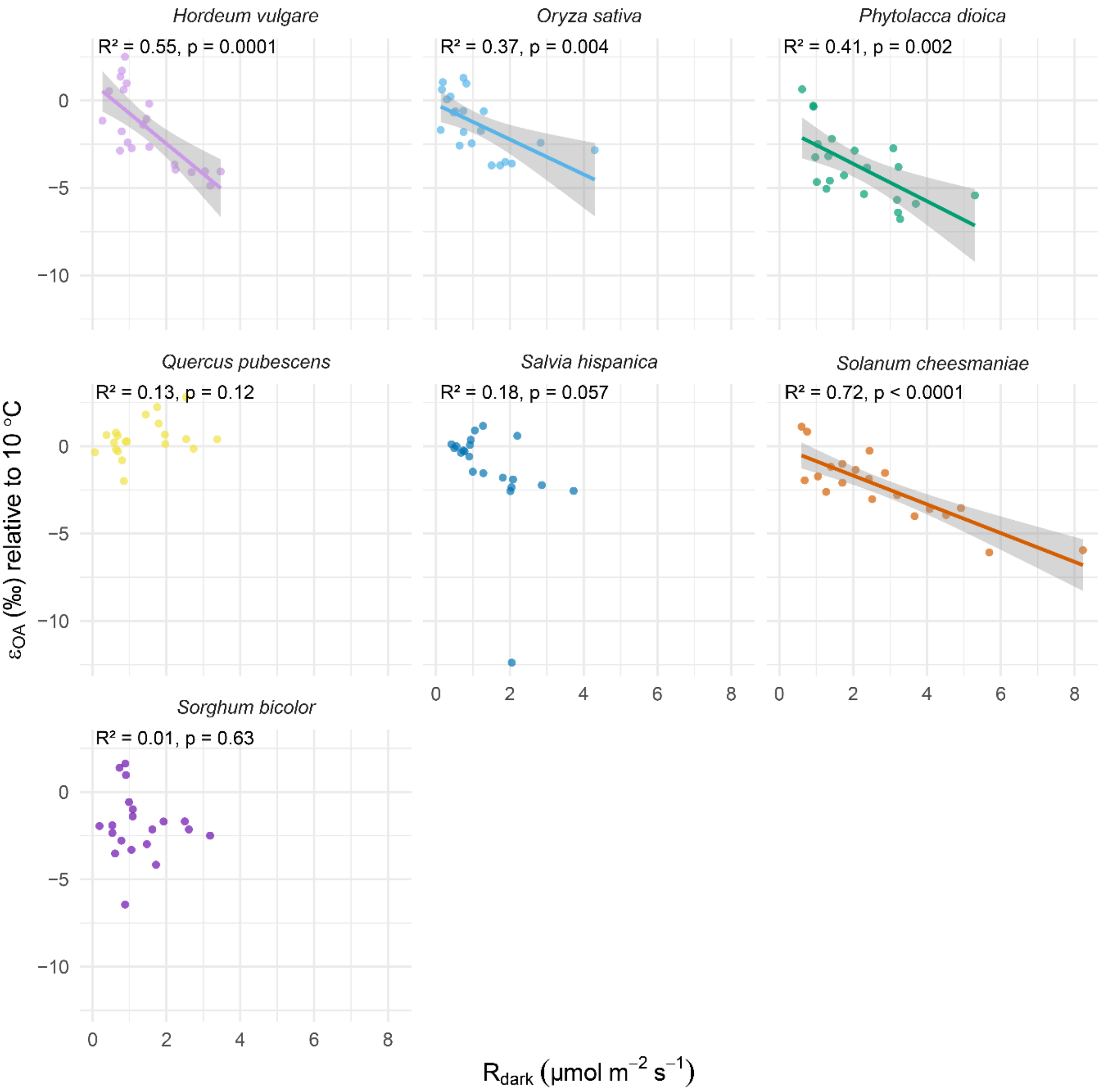

Supplementary Figure 8: Species-specific relative response ε_{OA} to R_{dark}. Only significant relationships are displayed with linear regressions. The shaded area represents the 95% confidence interval.

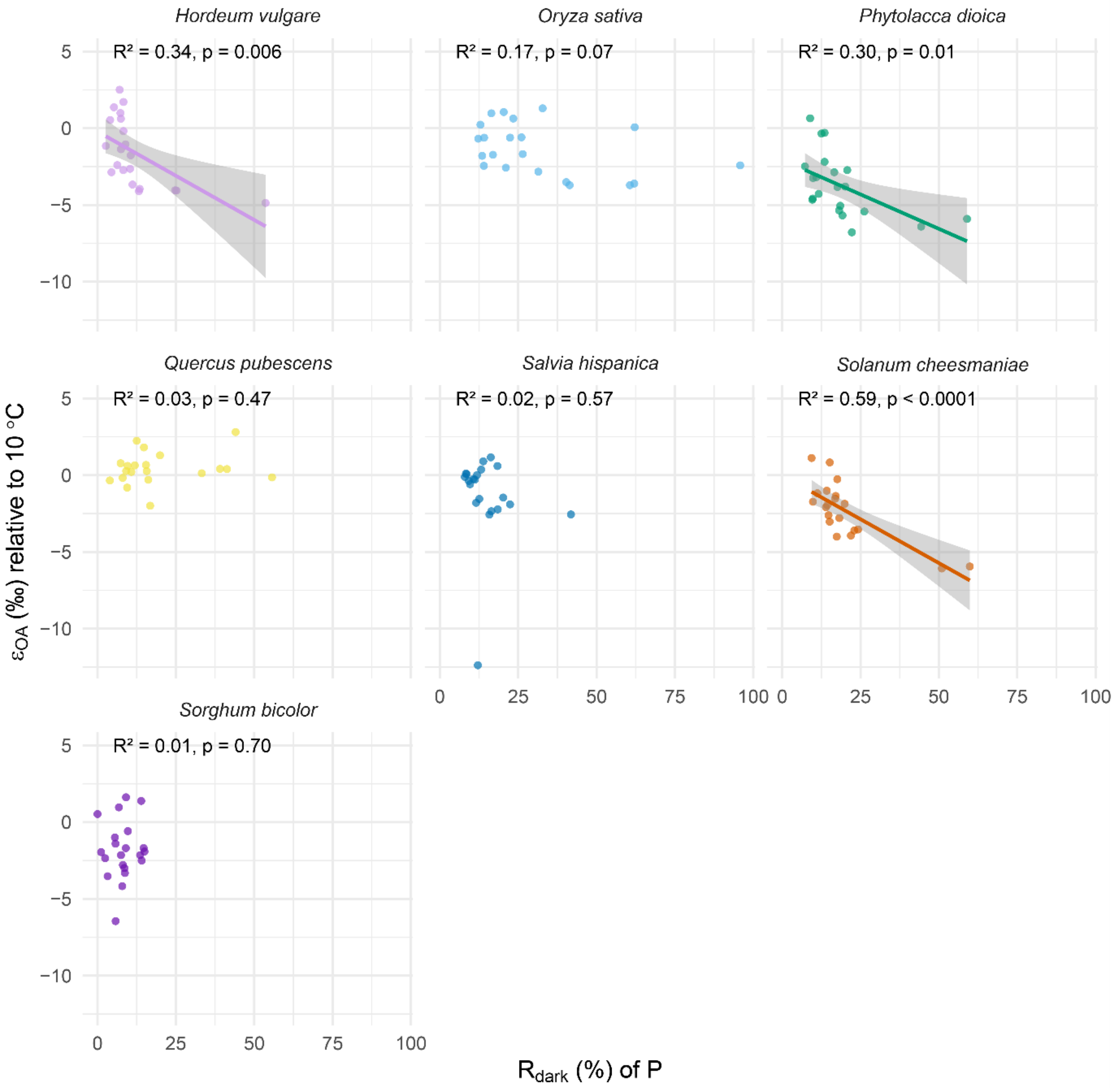

Supplementary Figure 9: Species-specific relative response ε_{OA} to R_{dark} in % of total photosynthesis (P). Only significant relationships are displayed with linear regressions. The shaded area represents the 95% confidence interval.

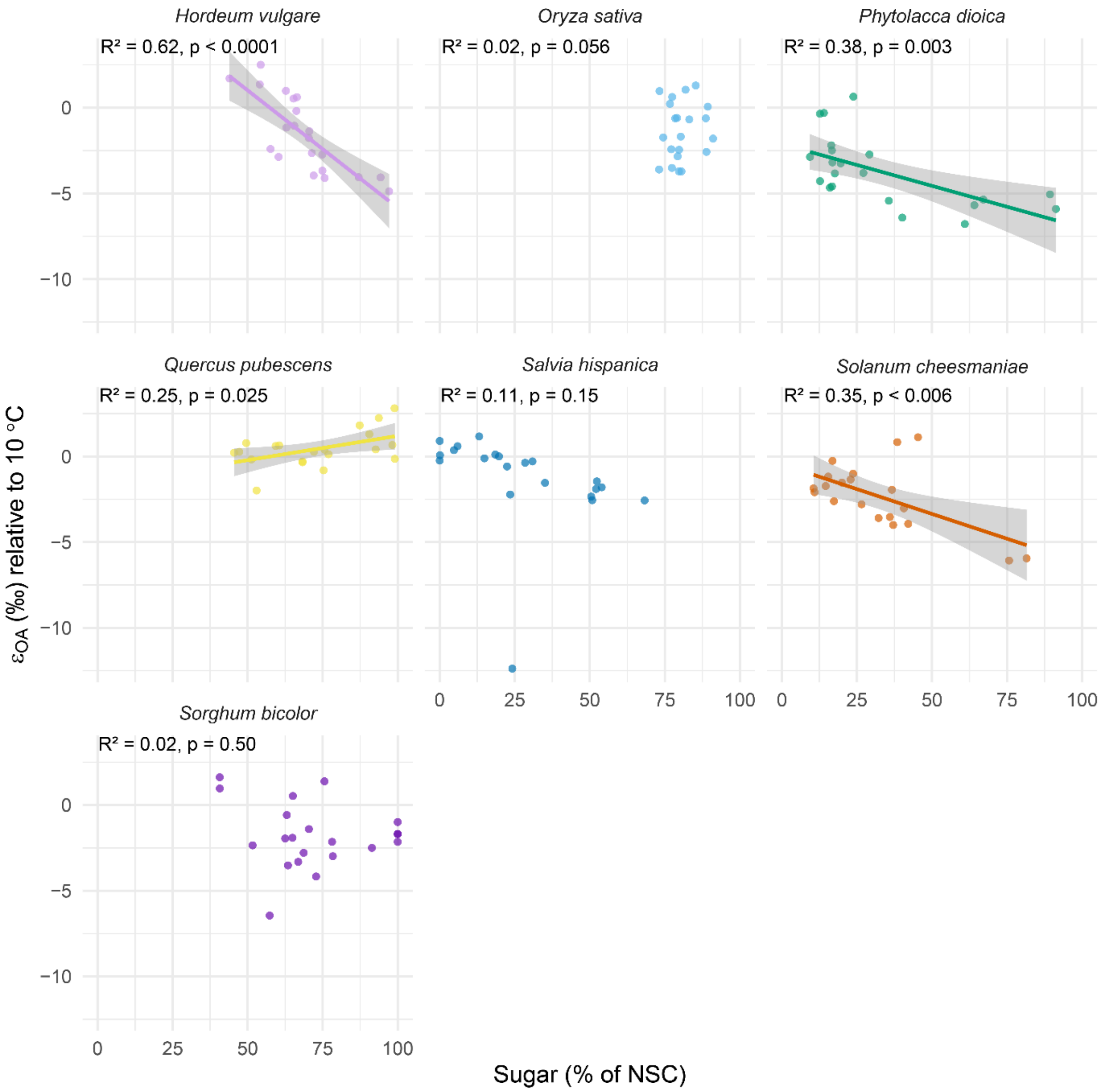

Supplementary Figure 10: Species-specific relative response ε_{OA} to the percentage sugar contributes to the whole leaf NSC pool. Only significant relationships are displayed with linear regressions. The shaded area represents the 95% confidence interval.

Supplementary Table 1: Type of photosynthesis (C_3 or C_4), net assimilation (A_{net}), dark respiration (R_{dark}), total photosynthesis (P; A_{net} + R_{dark}), stomatal conductance to water vapor (gsw) of the seven tested plant species.

Species	C_3 / C_4	temp [°C]	A_{net} [µmol m^{-2} s^{-1}]	R_{dark} [µmol m^{-2} s^{-1}]	P [µmol m^{-2} s^{-1}]	gsw [mol m^{-2} s^{-1}]
Hordeum vulgare	C_3	10	10.59	0.44	11.03	0.105
Hordeum vulgare	C_3	10	9.72	0.27	9.99	0.133
Hordeum vulgare	C_3	10	10.47	0.84	11.31	0.117
Hordeum vulgare	C_3	15	8.79	0.79	9.58	0.112
Hordeum vulgare	C_3	15	11.52	0.88	12.40	0.142
Hordeum vulgare	C_3	15	13.64	0.75	14.39	0.221
Hordeum vulgare	C_3	20	11.69	0.92	12.61	0.186
Hordeum vulgare	C_3	20	11.78	1.06	12.84	0.216
Hordeum vulgare	C_3	20	6.64	0.79	7.42	0.073
Hordeum vulgare	C_3	25	15.94	0.74	16.68	0.266
Hordeum vulgare	C_3	25	14.91	1.46	16.38	0.256
Hordeum vulgare	C_3	25	17.00	1.53	18.54	0.298
Hordeum vulgare	C_3	30	13.27	1.54	14.81	0.204
Hordeum vulgare	C_3	30	14.12	0.95	15.07	0.291
Hordeum vulgare	C_3	30	16.95	1.37	18.32	0.288
Hordeum vulgare	C_3	35	17.61	2.68	20.29	0.390
Hordeum vulgare	C_3	35	14.48	2.25	16.73	0.000
Hordeum vulgare	C_3	35	17.65	2.22	19.87	0.148
Hordeum vulgare	C_3	40	9.26	3.04	12.30	0.379
Hordeum vulgare	C_3	40	10.28	3.47	13.75	0.291
Hordeum vulgare	C_3	40	2.76	3.19	5.94	0.132
Oryza sativa	C_3	10	0.53	0.16	0.69	0.020
Oryza sativa	C_3	10	0.72	0.18	0.91	0.049
Oryza sativa	C_3	10	0.36	0.13	0.49	0.036
Oryza sativa	C_3	15	2.40	0.64	3.04	0.167
Oryza sativa	C_3	15	0.18	0.29	0.47	0.067
Oryza sativa	C_3	15	1.52	0.74	2.27	0.053
Oryza sativa	C_3	20	3.22	0.53	3.74	0.125
Oryza sativa	C_3	20	2.65	0.39	3.05	0.123
Oryza sativa	C_3	20	3.45	0.48	3.93	0.150
Oryza sativa	C_3	25	5.97	1.22	7.19	0.110
Oryza sativa	C_3	25	4.19	0.82	5.01	0.138
Oryza sativa	C_3	25	4.48	1.29	5.78	0.074
Oryza sativa	C_3	30	2.10	0.74	2.83	0.124
Oryza sativa	C_3	30	5.92	0.97	6.89	0.134
Oryza sativa	C_3	30	4.78	0.75	5.53	0.124
Oryza sativa	C_3	35	1.13	1.74	2.87	0.333
Oryza sativa	C_3	35	2.77	1.88	4.65	0.220
Oryza sativa	C_3	35	2.13	1.51	3.64	0.080
Oryza sativa	C_3	40	9.39	4.30	13.69	0.339
Oryza sativa	C_3	40	0.12	2.83	2.96	0.260
Oryza sativa	C_3	40	1.26	2.05	3.31	0.175

Phytolacca dioica	C_3	10	6.46	0.92	7.38	0.064
Phytolacca dioica	C_3	10	6.31	0.62	6.93	0.086
Phytolacca dioica	C_3	10	5.87	0.92	6.78	0.157
Phytolacca dioica	C_3	15	10.84	1.32	12.17	0.109
Phytolacca dioica	C_3	15	8.92	0.97	9.90	0.071
Phytolacca dioica	C_3	15	9.55	1.02	10.57	0.082
Phytolacca dioica	C_3	20	10.24	2.04	12.27	0.126
Phytolacca dioica	C_3	20	9.10	1.42	10.52	0.079
Phytolacca dioica	C_3	20	11.09	2.37	13.46	0.134
Phytolacca dioica	C_3	25	13.26	1.04	14.30	0.116
Phytolacca dioica	C_3	25	12.62	1.37	13.99	0.121
Phytolacca dioica	C_3	25	13.29	1.75	15.04	0.152
Phytolacca dioica	C_3	30	11.79	3.09	14.87	0.103
Phytolacca dioica	C_3	30	14.91	5.30	20.20	0.277
Phytolacca dioica	C_3	30	12.84	3.23	16.06	0.028
Phytolacca dioica	C_3	35	11.46	3.27	14.73	0.073
Phytolacca dioica	C_3	35	13.39	3.19	16.57	0.091
Phytolacca dioica	C_3	35	10.34	2.29	12.63	0.041
Phytolacca dioica	C_3	40	5.60	1.27	6.88	0.065
Phytolacca dioica	C_3	40	2.57	3.70	6.27	0.473
Phytolacca dioica	C_3	40	4.04	3.22	7.25	0.002
Quercus pubescens	C_3	10	1.55	0.06	1.62	0.004
Quercus pubescens	C_3	10	3.56	0.69	4.24	0.024
Quercus pubescens	C_3	10	2.78	0.38	3.15	0.020
Quercus pubescens	C_3	15	7.64	0.80	8.43	0.046
Quercus pubescens	C_3	15	4.20	0.85	5.04	0.026
Quercus pubescens	C_3	15	9.24	0.93	10.17	0.084
Quercus pubescens	C_3	20	4.79	0.58	5.37	0.034
Quercus pubescens	C_3	20	7.26	0.63	7.89	0.056
Quercus pubescens	C_3	20	4.82	0.90	5.72	0.038
Quercus pubescens	C_3	25	9.58	0.82	10.40	0.095
Quercus pubescens	C_3	25	6.39	0.68	7.07	0.056
Quercus pubescens	C_3	25	7.92	0.63	8.56	0.064
Quercus pubescens	C_3	30	7.19	1.79	8.98	0.037
Quercus pubescens	C_3	30	10.70	1.96	12.66	0.068
Quercus pubescens	C_3	30	8.27	1.44	9.71	0.061
Quercus pubescens	C_3	35	3.96	1.97	5.93	0.019
Quercus pubescens	C_3	35	12.21	1.75	13.96	0.087
Quercus pubescens	C_3	35	3.95	2.54	6.49	0.095
Quercus pubescens	C_3	40	3.21	2.53	5.74	0.000
Quercus pubescens	C_3	40	-0.55	2.73	4.91	0.000
Quercus pubescens	C_3	40	4.79	3.37	8.17	0.168

Salvia hispanica	C_3	10	4.63	0.42	5.05	0.036
Salvia hispanica	C_3	10	5.73	0.50	6.23	0.024
Salvia hispanica	C_3	10	4.13	0.55	4.68	0.043
Salvia hispanica	C_3	15	6.14	0.77	6.91	0.039
Salvia hispanica	C_3	15	6.66	0.68	7.34	0.039
Salvia hispanica	C_3	15	8.45	0.90	9.35	0.074
Salvia hispanica	C_3	20	6.22	0.94	7.16	0.050
Salvia hispanica	C_3	20	6.56	1.27	7.84	0.052
Salvia hispanica	C_3	20	9.78	2.20	11.99	0.134
Salvia hispanica	C_3	25	6.55	1.05	7.61	0.072
Salvia hispanica	C_3	25	9.85	0.92	10.78	0.090
Salvia hispanica	C_3	25	6.19	0.74	6.93	0.052
Salvia hispanica	C_3	30	8.90	1.28	10.19	0.069
Salvia hispanica	C_3	30	3.94	1.00	4.94	0.023
Salvia hispanica	C_3	30	13.84	1.81	15.65	0.171
Salvia hispanica	C_3	35	14.88	2.05	16.93	0.157
Salvia hispanica	C_3	35	10.74	2.02	12.75	0.109
Salvia hispanica	C_3	35	10.42	2.05	12.46	0.179
Salvia hispanica	C_3	40	5.18	3.73	8.91	0.136
Salvia hispanica	C_3	40	12.66	2.87	15.53	0.135
Salvia hispanica	C_3	40	7.23	2.09	9.32	0.083
Solanum cheesmaniae	C_3	10	5.83	0.60	6.43	0.065
Solanum cheesmaniae	C_3	10	4.08	0.69	4.77	0.066
Solanum cheesmaniae	C_3	10	4.21	0.75	4.96	0.059
Solanum cheesmaniae	C_3	15	7.31	1.27	8.58	0.094
Solanum cheesmaniae	C_3	15	11.06	1.40	12.45	0.136
Solanum cheesmaniae	C_3	15	10.03	2.06	12.10	0.112
Solanum cheesmaniae	C_3	20	9.75	2.42	12.17	0.107
Solanum cheesmaniae	C_3	20	10.53	1.71	12.23	0.117
Solanum cheesmaniae	C_3	20	11.46	2.44	13.90	0.132
Solanum cheesmaniae	C_3	25	10.30	1.71	12.01	0.106
Solanum cheesmaniae	C_3	25	9.63	1.05	10.67	0.100
Solanum cheesmaniae	C_3	25	14.07	2.86	16.93	0.167
Solanum cheesmaniae	C_3	30	14.33	3.19	17.52	0.124
Solanum cheesmaniae	C_3	30	15.41	4.92	20.33	0.154
Solanum cheesmaniae	C_3	30	14.10	2.51	16.61	0.134
Solanum cheesmaniae	C_3	35	17.40	3.66	21.06	0.346
Solanum cheesmaniae	C_3	35	16.09	4.52	20.61	0.170
Solanum cheesmaniae	C_3	35	13.62	4.07	17.69	0.092
Solanum cheesmaniae	C_3	40	-2.70	8.22	13.74	0.132
Solanum cheesmaniae	C_3	40	-0.22	5.69	11.16	0.000

Sorghum bicolor	C_4	10	3.03	0.53	3.56	0.010
Sorghum bicolor	C_4	10	4.49	0.73	5.22	0.039
Sorghum bicolor	C_4	10	2.58		2.58	0.039
Sorghum bicolor	C_4	15	9.21	0.99	10.19	0.108
Sorghum bicolor	C_4	15	8.84	0.78	9.62	0.125
Sorghum bicolor	C_4	15	8.87	0.89	9.75	0.054
Sorghum bicolor	C_4	20	14.33	0.88	15.21	0.068
Sorghum bicolor	C_4	20	11.00	1.05	12.06	0.056
Sorghum bicolor	C_4	20	12.34	0.90	13.24	0.065
Sorghum bicolor	C_4	25	16.07	0.19	16.26	0.093
Sorghum bicolor	C_4	25	21.52	0.54	22.05	0.120
Sorghum bicolor	C_4	25	18.31	0.61	18.92	0.111
Sorghum bicolor	C_4	30	19.36	1.92	21.28	0.104
Sorghum bicolor	C_4	30	19.97	1.62	21.59	0.160
Sorghum bicolor	C_4	30	18.55	1.09	19.64	0.126
Sorghum bicolor	C_4	35	15.66	1.47	17.13	0.111
Sorghum bicolor	C_4	35	17.84	1.09	18.93	0.039
Sorghum bicolor	C_4	35	20.06	1.72	21.78	0.210
Sorghum bicolor	C_4	40	16.57	2.61	19.18	0.176
Sorghum bicolor	C_4	40	14.46	2.50	16.96	0.266
Sorghum bicolor	C_4	40	19.42	3.18	22.60	0.194

Supplementary Table 2: Leaf chlorophyll fluorescence measurements, the ratio of minimum to maximum fluorescence (FvFm), Photosystem II efficiency (ΦPSII), electron transport rate (ETR), and the non-photochemical quenching (NPQ) of the seven tested plant species.

Species	temp [°C]	Fv/Fm	ΦPSII	ETR [µmol s^{-1}]	NPQ
Hordeum vulgare	10	0.79	0.41	52.31	0.68
Hordeum vulgare	10	0.81	0.38	47.61	1.35
Hordeum vulgare	10	0.77	0.40	50.13	0.92
Hordeum vulgare	15	0.79	0.47	59.40	1.01
Hordeum vulgare	15	0.79	0.51	64.01	0.78
Hordeum vulgare	15	0.80	0.53	67.34	0.65
Hordeum vulgare	20	0.81	0.54	68.42	0.49
Hordeum vulgare	20	0.80	0.55	69.32	0.55
Hordeum vulgare	20	0.81	0.49	61.55	0.49
Hordeum vulgare	25	0.81	0.55	93.55	0.42
Hordeum vulgare	25	0.78	0.55	92.66	0.38
Hordeum vulgare	25	0.80	0.57	96.32	0.33
Hordeum vulgare	30	0.80	0.56	94.82	0.34
Hordeum vulgare	30	0.78	0.61	103.56	0.13
Hordeum vulgare	30	0.79	0.58	98.32	0.23
Hordeum vulgare	35	0.76	0.57	107.24	0.21
Hordeum vulgare	35	0.73	0.49	93.23	0.27
Hordeum vulgare	35	0.76	0.57	108.16	0.17
Hordeum vulgare	40	0.76	0.42	80.03	0.77
Hordeum vulgare	40	0.77	0.49	92.46	0.53
Hordeum vulgare	40	0.55	0.21	40.30	0.68

Oryza sativa	10	0.71	0.03	3.69	0.78
Oryza sativa	10	0.68	0.07	8.81	1.27
Oryza sativa	10	0.76	0.02	2.90	0.93
Oryza sativa	15	0.72	0.14	17.61	0.83
Oryza sativa	15	0.59	0.06	7.42	0.58
Oryza sativa	15	0.68	0.16	19.99	1.08
Oryza sativa	20	0.72	0.20	24.68	0.77
Oryza sativa	20	0.72	0.17	21.83	0.90
Oryza sativa	20	0.76	0.18	22.73	1.58
Oryza sativa	25	0.76	0.37	63.03	1.46
Oryza sativa	25	0.68	0.21	35.26	1.11
Oryza sativa	25	0.72	0.30	51.30	0.93
Oryza sativa	30	0.68	0.20	34.20	1.12
Oryza sativa	30	0.54	0.26	43.79	0.73
Oryza sativa	30	0.58	0.19	32.64	0.80
Oryza sativa	35	0.53	0.11	21.15	1.39
Oryza sativa	35	0.64	0.12	23.35	0.66
Oryza sativa	35	0.60	0.11	21.44	0.89
Oryza sativa	40	0.78	0.41	77.40	0.69
Oryza sativa	40	0.46	0.04	7.39	0.93
Oryza sativa	40	0.53	0.07	12.45	0.91
Phytolacca dioica	10	0.76	0.22	28.04	1.59
Phytolacca dioica	10	0.76	0.23	28.51	1.41
Phytolacca dioica	10	0.66	0.21	26.65	1.18
Phytolacca dioica	15	0.78	0.50	63.19	0.98
Phytolacca dioica	15	0.77	0.48	60.21	1.02
Phytolacca dioica	15	0.79	0.46	58.29	0.91
Phytolacca dioica	20	0.81	0.58	73.00	0.57
Phytolacca dioica	20	0.83	0.59	75.18	0.74
Phytolacca dioica	20	0.79	0.60	75.60	0.48
Phytolacca dioica	25	0.82	0.61	102.58	0.33
Phytolacca dioica	25	0.83	0.61	103.48	0.34
Phytolacca dioica	25	0.82	0.61	102.85	0.38
Phytolacca dioica	30	0.83	0.63	106.99	0.42
Phytolacca dioica	30	0.82	0.64	108.05	0.32
Phytolacca dioica	30	0.83	0.64	108.46	0.50
Phytolacca dioica	35	0.82	0.59	112.45	0.65
Phytolacca dioica	35	0.82	0.58	110.84	0.57
Phytolacca dioica	35	0.83	0.57	108.59	0.79
Phytolacca dioica	40	0.76	0.28	53.53	1.42
Phytolacca dioica	40	0.59	0.22	40.98	0.97
Phytolacca dioica	40	0.74	0.30	56.76	1.48

Quercus pubescens	10	0.77	0.19	24.23	1.47
Quercus pubescens	10	0.74	0.21	26.70	1.19
Quercus pubescens	10	0.73	0.12	15.01	0.60
Quercus pubescens	15	0.79	0.54	68.06	0.69
Quercus pubescens	15	0.75	0.42	53.37	0.76
Quercus pubescens	15	0.78	0.47	59.66	0.55
Quercus pubescens	20	0.80	0.51	64.58	0.73
Quercus pubescens	20	0.80	0.55	69.15	0.47
Quercus pubescens	20	0.79	0.35	44.57	0.46
Quercus pubescens	25	0.80	0.53	90.00	0.40
Quercus pubescens	25	0.80	0.50	84.47	0.68
Quercus pubescens	25	0.81	0.39	66.29	0.35
Quercus pubescens	30	0.80	0.57	96.30	0.59
Quercus pubescens	30	0.78	0.53	89.97	0.16
Quercus pubescens	30	0.80	0.54	91.58	0.31
Quercus pubescens	35	0.75	0.38	72.71	0.59
Quercus pubescens	35	0.75	0.52	98.89	0.29
Quercus pubescens	35	0.75	0.33	61.84	0.45
Quercus pubescens	40	0.34	0.19	36.90	0.09
Quercus pubescens	40	0.53	0.03	6.17	1.11
Quercus pubescens	40	0.72	0.39	73.69	0.84
Salvia hispanica	10	0.77	0.16	20.68	1.97
Salvia hispanica	10	0.72	0.25	31.48	1.07
Salvia hispanica	10	0.80	0.16	20.23	1.45
Salvia hispanica	15	0.82	0.33	41.15	1.58
Salvia hispanica	15	0.82	0.41	51.34	1.36
Salvia hispanica	15	0.81	0.48	61.13	1.14
Salvia hispanica	20	0.81	0.51	64.08	0.77
Salvia hispanica	20	0.80	0.50	63.10	0.98
Salvia hispanica	20	0.79	0.55	69.26	0.71
Salvia hispanica	25	0.79	0.42	71.60	1.20
Salvia hispanica	25	0.80	0.54	90.99	0.61
Salvia hispanica	25	0.80	0.52	87.18	0.69
Salvia hispanica	30	0.80	0.57	95.60	0.57
Salvia hispanica	30	0.78	0.45	76.39	1.17
Salvia hispanica	30	0.80	0.63	105.73	0.33
Salvia hispanica	35	0.79	0.63	119.83	0.27
Salvia hispanica	35	0.80	0.58	109.08	0.51
Salvia hispanica	35	0.76	0.57	108.06	0.24
Salvia hispanica	40	0.73	0.43	81.59	0.94
Salvia hispanica	40	0.74	0.57	108.38	0.21
Salvia hispanica	40	0.75	0.50	95.01	0.65

Solanum cheesmaniae	10	0.79	0.24	30.25	1.47
Solanum cheesmaniae	10	0.69	0.15	18.35	1.02
Solanum cheesmaniae	10	0.71	0.15	19.20	0.96
Solanum cheesmaniae	15	0.82	0.42	53.67	0.82
Solanum cheesmaniae	15	0.83	0.52	66.10	0.75
Solanum cheesmaniae	15	0.81	0.51	64.27	0.61
Solanum cheesmaniae	20	0.81	0.61	76.71	0.39
Solanum cheesmaniae	20	0.83	0.57	72.45	0.44
Solanum cheesmaniae	20	0.82	0.65	82.70	0.26
Solanum cheesmaniae	25	0.84	0.49	83.44	0.87
Solanum cheesmaniae	25	0.82	0.46	77.06	0.99
Solanum cheesmaniae	25	0.83	0.61	102.31	0.38
Solanum cheesmaniae	30	0.83	0.63	106.24	0.31
Solanum cheesmaniae	30	0.82	0.69	116.98	0.22
Solanum cheesmaniae	30	0.84	0.63	106.30	0.32
Solanum cheesmaniae	35	0.80	0.65	122.64	0.29
Solanum cheesmaniae	35	0.81	0.65	123.74	0.24
Solanum cheesmaniae	35	0.81	0.61	114.95	0.37
Solanum cheesmaniae	40	0.68	0.19	35.14	1.19
Solanum cheesmaniae	40	0.70	0.22	41.96	1.19
Sorghum bicolor	10	0.75	0.12	15.01	1.56
Sorghum bicolor	10	0.72	0.19	23.63	1.30
Sorghum bicolor	10	0.75	0.11	13.37	1.12
Sorghum bicolor	15	0.76	0.38	47.57	1.31
Sorghum bicolor	15	0.76	0.37	46.49	1.18
Sorghum bicolor	15	0.77	0.39	48.77	1.31
Sorghum bicolor	20	0.77	0.58	72.76	0.47
Sorghum bicolor	20	0.77	0.53	67.07	0.69
Sorghum bicolor	20	0.77	0.53	67.18	0.62
Sorghum bicolor	25	0.78	0.48	80.58	0.72
Sorghum bicolor	25	0.79	0.56	94.41	0.52
Sorghum bicolor	25	0.78	0.52	87.52	0.56
Sorghum bicolor	30	0.77	0.59	99.91	0.41
Sorghum bicolor	30	0.77	0.60	101.49	0.34
Sorghum bicolor	30	0.77	0.54	91.81	0.45
Sorghum bicolor	35	0.75	0.44	84.10	0.75
Sorghum bicolor	35	0.73	0.46	87.07	0.48
Sorghum bicolor	35	0.76	0.50	94.35	0.62
Sorghum bicolor	40	0.73	0.46	87.11	0.57
Sorghum bicolor	40	0.76	0.43	81.18	0.93
Sorghum bicolor	40	0.74	0.53	100.61	0.50

Supplementary Table 3: Amount of non-structural carbohydrates (NSC), sugar, starch, per 100 mg leaf dry mass, the contibrution in % of sugar and starch to the total leaf NSC, and the ratio of leaf sugar to leaf starch of the seven tested species.

Species	temp [°C]	NSC [mg 100 mg^{-1}]	Sugar [mg 100 mg^{-1}]	Starch [mg 100 mg^{-1}]	Sugar [% of NSC]	Starch [% of NSC]
Hordeum vulgare	10	8.29	5.41	2.88	65	35
Hordeum vulgare	10	7.17	4.52	2.66	63	37
Hordeum vulgare	10	5.36	3.56	1.80	66	34
Hordeum vulgare	15	2.21	0.97	1.24	44	56
Hordeum vulgare	15	3.57	1.94	1.63	54	46
Hordeum vulgare	15	3.56	1.92	1.63	54	46
Hordeum vulgare	20	8.14	5.11	3.03	63	37
Hordeum vulgare	20	9.67	7.24	2.43	75	25
Hordeum vulgare	20	10.18	7.17	3.01	70	30
Hordeum vulgare	25	5.85	3.53	2.32	60	40
Hordeum vulgare	25	6.27	4.11	2.16	66	34
Hordeum vulgare	25	5.57	3.69	1.88	66	34
Hordeum vulgare	30	4.94	3.53	1.41	71	29
Hordeum vulgare	30	4.83	2.78	2.04	58	42
Hordeum vulgare	30	4.97	3.51	1.46	71	29
Hordeum vulgare	35	9.86	7.46	2.40	76	24
Hordeum vulgare	35	8.03	5.79	2.25	72	28
Hordeum vulgare	35	7.51	5.63	1.88	75	25
Hordeum vulgare	40	8.18	7.12	1.06	87	13
Hordeum vulgare	40	5.35	5.05	0.31	94	6
Hordeum vulgare	40	8.15	7.91	0.23	97	3
Oryza sativa	10	11.16	8.65	2.52	77	23
Oryza sativa	10	10.83	8.87	1.96	82	18
Oryza sativa	10	14.72	11.82	2.90	80	20
Oryza sativa	15	16.79	14.92	1.87	89	11
Oryza sativa	15	18.51	16.53	1.98	89	11
Oryza sativa	15	17.40	14.84	2.56	85	15
Oryza sativa	20	12.67	9.94	2.73	78	22
Oryza sativa	20	13.36	10.24	3.11	77	23
Oryza sativa	20	14.02	11.66	2.36	83	17
Oryza sativa	25	13.00	9.68	3.32	74	26
Oryza sativa	25	13.00	9.52	3.48	73	27
Oryza sativa	25	11.03	9.78	1.25	89	11
Oryza sativa	30	9.07	7.17	1.90	79	21
Oryza sativa	30	9.76	7.78	1.97	80	20
Oryza sativa	30	7.85	7.15	0.70	91	9
Oryza sativa	35	7.16	5.71	1.45	80	20
Oryza sativa	35	7.54	5.83	1.71	77	23
Oryza sativa	35	10.11	8.14	1.97	81	19
Oryza sativa	40	7.47	5.92	1.55	79	21
Oryza sativa	40	6.00	4.63	1.37	77	23
Oryza sativa	40	6.45	4.72	1.74	73	27

Phytolacca dioica	10	16.68	2.12	14.57	13	87
Phytolacca dioica	10	9.11	2.17	6.94	24	76
Phytolacca dioica	10	15.12	2.12	13.01	14	86
Phytolacca dioica	15	14.50	2.44	12.06	17	83
Phytolacca dioica	15	12.20	2.39	9.81	20	80
Phytolacca dioica	15	10.35	1.66	8.70	16	84
Phytolacca dioica	20	17.68	1.65	16.02	9	91
Phytolacca dioica	20	19.32	3.18	16.15	16	84
Phytolacca dioica	20	13.53	2.39	11.15	18	82
Phytolacca dioica	25	10.94	1.82	9.11	17	83
Phytolacca dioica	25	10.95	1.83	9.12	17	83
Phytolacca dioica	25	16.12	2.05	14.07	13	87
Phytolacca dioica	30	8.25	2.41	5.85	29	71
Phytolacca dioica	30	9.16	3.26	5.89	36	64
Phytolacca dioica	30	10.62	2.88	7.74	27	73
Phytolacca dioica	35	7.02	4.28	2.74	61	39
Phytolacca dioica	35	2.96	1.90	1.06	64	36
Phytolacca dioica	35	5.13	3.44	1.69	67	33
Phytolacca dioica	40	5.00	4.47	0.53	89	11
Phytolacca dioica	40	4.28	3.91	0.37	91	9
Phytolacca dioica	40	8.60	3.45	5.15	40	60
Quercus pubescens	10	10.31	7.04	3.27	68	32
Quercus pubescens	10	9.79	6.69	3.11	68	32
Quercus pubescens	10	14.56	8.81	5.75	61	39
Quercus pubescens	15	9.00	6.78	2.22	75	25
Quercus pubescens	15	14.34	7.60	6.74	53	47
Quercus pubescens	15	12.29	8.86	3.43	72	28
Quercus pubescens	20	13.59	6.18	7.40	46	54
Quercus pubescens	20	13.77	7.06	6.71	51	49
Quercus pubescens	20	16.66	7.87	8.79	47	53
Quercus pubescens	25	6.27	5.03	1.24	80	20
Quercus pubescens	25	10.30	6.11	4.19	59	41
Quercus pubescens	25	12.02	5.95	6.06	50	50
Quercus pubescens	30	9.65	8.74	0.91	91	9
Quercus pubescens	30	8.94	8.79	0.16	98	2
Quercus pubescens	30	10.40	9.08	1.32	87	13
Quercus pubescens	35	5.99	4.61	1.38	77	23
Quercus pubescens	35	7.01	6.57	0.44	94	6
Quercus pubescens	35	8.02	7.43	0.59	93	7
Quercus pubescens	40	4.15	4.11	0.04	99	1
Quercus pubescens	40	5.37	5.31	0.05	99	1
Quercus pubescens	40	8.12	6.15	1.98	76	24

Salvia hispanica	10	15.47	2.86	12.61	19	81
Salvia hispanica	10	15.39	2.29	13.11	15	85
Salvia hispanica	10	21.33	4.21	17.11	20	80
Salvia hispanica	15	10.83	3.35	7.48	31	69
Salvia hispanica	15	8.86	2.52	6.34	28	72
Salvia hispanica	15	14.49	3.25	11.24	22	78
Salvia hispanica	20	6.61	0.31	6.30	5	95
Salvia hispanica	20	4.04	0.53	3.51	13	87
Salvia hispanica	20	5.31	0.32	5.00	6	94
Salvia hispanica	25	7.29	0.00	7.29	0	100
Salvia hispanica	25	3.91	0.00	3.91	0	100
Salvia hispanica	25	3.87	0.00	3.87	0	100
Salvia hispanica	30	5.20	1.83	3.38	35	65
Salvia hispanica	30	2.66	1.39	1.27	52	48
Salvia hispanica	30	3.14	1.70	1.45	54	46
Salvia hispanica	35	3.86	0.93	2.93	24	76
Salvia hispanica	35	2.25	1.54	0.72	68	32
Salvia hispanica	35	3.19	1.61	1.58	50	50
Salvia hispanica	40	6.02	3.06	2.96	51	49
Salvia hispanica	40	12.41	2.91	9.49	23	77
Salvia hispanica	40	4.75	2.47	2.28	52	48
Solanum cheesmaniae	10	22.31	10.12	12.19	45	55
Solanum cheesmaniae	10	21.43	7.85	13.57	37	63
Solanum cheesmaniae	10	24.25	9.32	14.93	38	62
Solanum cheesmaniae	15	17.98	3.12	14.86	17	83
Solanum cheesmaniae	15	17.97	2.77	15.20	15	85
Solanum cheesmaniae	15	23.85	5.46	18.39	23	77
Solanum cheesmaniae	20	29.27	3.09	26.19	11	89
Solanum cheesmaniae	20	25.67	2.80	22.87	11	89
Solanum cheesmaniae	20	19.52	3.29	16.23	17	83
Solanum cheesmaniae	25	17.68	4.19	13.49	24	76
Solanum cheesmaniae	25	23.54	3.44	20.11	15	85
Solanum cheesmaniae	25	15.11	3.02	12.09	20	80
Solanum cheesmaniae	30	14.74	3.91	10.83	27	73
Solanum cheesmaniae	30	27.49	9.89	17.60	36	64
Solanum cheesmaniae	30	16.99	6.91	10.09	41	59
Solanum cheesmaniae	35	28.65	10.62	18.03	37	63
Solanum cheesmaniae	35	24.68	10.38	14.30	42	58
Solanum cheesmaniae	35	26.10	8.41	17.69	32	68
Solanum cheesmaniae	40	15.39	12.55	2.84	82	18
Solanum cheesmaniae	40	16.46	12.45	4.00	76	24

Sorghum bicolor	10	17.68	11.49	6.20	65	35
Sorghum bicolor	10	15.31	11.57	3.74	76	24
Sorghum bicolor	10	16.85	10.96	5.88	65	35
Sorghum bicolor	15	13.82	8.71	5.11	63	37
Sorghum bicolor	15	11.24	7.71	3.52	69	31
Sorghum bicolor	15	19.35	7.88	11.47	41	59
Sorghum bicolor	20	9.63	5.53	4.11	57	43
Sorghum bicolor	20	12.87	8.60	4.26	67	33
Sorghum bicolor	20	21.14	8.61	12.53	41	59
Sorghum bicolor	25	6.37	3.98	2.39	63	37
Sorghum bicolor	25	5.73	2.96	2.77	52	48
Sorghum bicolor	25	7.34	4.66	2.68	63	37
Sorghum bicolor	30	10.76	10.89	0.00	100	0
Sorghum bicolor	30	10.82	11.64	0.00	100	0
Sorghum bicolor	30	11.92	12.33	0.00	100	0
Sorghum bicolor	35	5.36	4.20	1.16	78	22
Sorghum bicolor	35	4.30	3.03	1.27	70	30
Sorghum bicolor	35	5.55	4.04	1.51	73	27
Sorghum bicolor	40	5.99	4.68	1.31	78	22
Sorghum bicolor	40	4.86	4.93	0.00	100	0
Sorghum bicolor	40	5.58	5.10	0.48	91	9

Supplementary Table 4: p-values of the Mann-Whitney U (across all species) and the Welche two Sample t-test (within each species), comparing the NSC concentration in mg 100 mg^{-1} and the percentage starch contributes to the total leaf NSC pool (% Starch) at 10 and 15 °C with the corresponding values at 35 and 40 °C.

p-values of comparing measured values at 10 and 15 °C *vs*. 35 and 40 °C

**Shapiro-Wilk normality tests**	**across all species**	***Hordeum vulgare***	***Oryza sativa***	***Phytolacca dioica***	***Quercus pubescens***	***Salvia hispanica***	***Solanum cheesmaniae***	***Sorghum bicolor***
NSC (mg 100 mg^{-1}) @ 10 & 15 °C	0.773	0.630	0.287	0.768	0.324	0.692	0.211	0.962
NSC (mg 100 mg^{-1}) @ 35 & 40 °C	< 0.001	0.389	0.198	0.812	0.642	0.083	0.276	0.676
% Starch @ 10 & 15 °C	0.003	0.377	0.604	0.729	0.579	0.717	0.383	0.106
% Starch @ 35 & 40 °C	0.005	0.259	0.332	0.516	0.080	0.211	0.142	0.337
**Mann-Whitney U test**								
NSC (mg 100 mg^{-1})	< 0.001	n.a.	n.a.	n.a.	n.a.	n.a.	n.a.	n.a.
% Starch	< 0.001	n.a.	n.a.	n.a.	n.a.	n.a.	n.a.	n.a.
**Welch Two Sample t-test**								
NSC (mg 100 mg^{-1})	n.a.	0.036	0.001	0.001	0.002	0.003	0.753	< 0.001
% Starch	n.a.	0.001	0.028	0.001	0.002	0.025	0.079	0.019

Supplementary Table 5: $\delta^{18}O$, $\delta^{2}H$ of leaf water ($\delta^{18}O_{LW}$, $\delta^{2}H_{LW}$) and leaf sugar ($\delta^{18}O_{LS}$, $\delta^{2}H_{LS}$), as well as the biological fractionation factors (ε_{OA}, ε_{HA}) between leaf water and leaf sugar of the seven tested species.

Species	temp [°C]	$\delta^{18}O_{LW}$	$\delta^{18}O_{LS}$	ε_{OA}	$\delta^{2}H_{LW}$	$\delta^{2}H_{LS}$	ε_{HA}
Hordeum vulgare	10	-0.44	29.34	29.8	-40.52	-141.75	-101.23
Hordeum vulgare	10	0.50	28.59	28.1	-38.22	-146.26	-108.04
Hordeum vulgare	10	1.92	31.78	29.9	-38.53	-116.61	-78.08
Hordeum vulgare	15	-1.17	29.78	31.0	-45.96	-115.42	-69.46
Hordeum vulgare	15	-1.52	30.24	31.8	-49.35	-130.19	-80.84
Hordeum vulgare	15	-1.97	28.64	30.6	-47.85	-135.67	-87.82
Hordeum vulgare	20	-1.74	28.49	30.2	-48.27	-150.83	-102.56
Hordeum vulgare	20	0.20	26.71	26.5	-41.40	-162.62	-121.22
Hordeum vulgare	20	0.00	27.48	27.5	-42.49	-156.01	-113.52
Hordeum vulgare	25	0.47	26.85	26.4	-16.48	-146.45	-129.97
Hordeum vulgare	25	-2.34	25.86	28.2	-26.26	-148.65	-122.39
Hordeum vulgare	25	-2.40	26.65	29.1	-34.80	-143.52	-108.72
Hordeum vulgare	30	-0.58	26.02	26.6	-29.82	-117.69	-87.87
Hordeum vulgare	30	-1.37	25.47	26.8	-31.51	-108.23	-76.72
Hordeum vulgare	30	-2.04	25.83	27.9	-32.84	-101.26	-68.42
Hordeum vulgare	35	-3.12	22.03	25.1	-36.65	-108.48	-71.83
Hordeum vulgare	35	-3.41	21.88	25.3	-35.74	-102.14	-66.40
Hordeum vulgare	35	-3.09	22.48	25.6	-34.11	-103.34	-69.23
Hordeum vulgare	40	-3.99	21.20	25.2	-38.02	-90.18	-52.16
Hordeum vulgare	40	-4.18	21.00	25.2	-39.99	-75.19	-35.20
Hordeum vulgare	40	-3.47	20.90	24.4	-37.62	-82.85	-45.23

Oryza sativa	10	0.20	32.05	31.84	-41.25	-68.97	-27.72
Oryza sativa	10	0.36	32.63	32.27	-42.05	-64.93	-22.88
Oryza sativa	10	1.98	31.51	29.53	-33.30	-68.07	-34.77
Oryza sativa	15	1.74	30.37	28.64	-43.77	-99.38	-55.61
Oryza sativa	15	0.00	31.28	31.28	-46.87	-100.79	-53.92
Oryza sativa	15	1.00	33.51	32.51	-44.44	-68.68	-24.24
Oryza sativa	20	2.48	33.08	30.60	-40.94	-86.42	-45.48
Oryza sativa	20	3.57	35.01	31.44	-36.01	-65.65	-29.64
Oryza sativa	20	1.32	31.86	30.53	-44.17	-86.90	-42.73
Oryza sativa	25	1.55	31.03	29.48	-27.63	-76.95	-49.32
Oryza sativa	25	-1.50	30.68	32.19	-38.46	-67.30	-28.84
Oryza sativa	25	0.16	30.76	30.60	-31.35	-85.58	-54.23
Oryza sativa	30	-0.43	30.19	30.62	-36.28	-23.66	12.62
Oryza sativa	30	0.03	28.80	28.76	-33.83	-43.48	-9.65
Oryza sativa	30	-0.84	28.58	29.42	-35.63	-13.07	22.56
Oryza sativa	35	-1.91	25.60	27.50	-39.09	-20.40	18.69
Oryza sativa	35	-2.40	25.30	27.70	-41.12	-17.59	23.53
Oryza sativa	35	-2.35	25.16	27.50	-42.78	-52.00	-9.22
Oryza sativa	40	-4.15	24.23	28.38	-41.47	-8.50	32.97
Oryza sativa	40	-4.18	24.62	28.79	-41.58	-13.57	28.01
Oryza sativa	40	-3.86	23.75	27.61	-38.21	-10.59	27.62
Phytolacca dioica	10	1.06	31.25	30.19	-31.01	-130.41	-99.40
Phytolacca dioica	10	-0.04	31.14	31.18	-34.08	-118.26	-84.18
Phytolacca dioica	10	0.01	30.24	30.23	-30.65	-122.64	-91.99
Phytolacca dioica	15	4.82	32.17	27.35	-23.15	-138.48	-115.33
Phytolacca dioica	15	5.50	32.78	27.29	-22.88	-128.11	-105.23
Phytolacca dioica	15	4.84	30.71	25.87	-28.08	-120.74	-92.66
Phytolacca dioica	20	1.68	29.35	27.67	-31.44	-124.05	-92.61
Phytolacca dioica	20	3.23	31.57	28.34	-27.92	-131.06	-103.14
Phytolacca dioica	20	2.50	29.20	26.70	-32.32	-141.65	-109.33
Phytolacca dioica	25	-0.20	27.85	28.05	-25.80	-126.22	-100.42
Phytolacca dioica	25	0.75	26.69	25.95	-26.49	-121.83	-95.34
Phytolacca dioica	25	1.97	28.22	26.25	-21.06	-134.64	-113.58
Phytolacca dioica	30	-2.15	25.65	27.80	-32.69	-117.48	-84.79
Phytolacca dioica	30	-0.11	24.99	25.11	-27.83	-126.22	-98.39
Phytolacca dioica	30	-1.32	25.40	26.73	-29.38	-133.70	-104.32
Phytolacca dioica	35	-0.81	22.94	23.75	-31.37	-133.31	-101.94
Phytolacca dioica	35	-2.67	22.18	24.85	-43.85	-112.06	-68.21
Phytolacca dioica	35	-1.86	23.32	25.18	-37.44	-121.67	-84.23
Phytolacca dioica	40	0.11	25.59	25.48	-30.16	-80.74	-50.58
Phytolacca dioica	40	-0.20	24.43	24.63	-33.72	-98.08	-64.36
Phytolacca dioica	40	0.25	24.37	24.12	-33.86	-79.75	-45.89

Quercus pubescens	10	7.13	31.30	24.18	-30.16	-98.37	-68.21
Quercus pubescens	10	5.76	29.99	24.22	-29.57	-103.92	-74.35
Quercus pubescens	10	5.65	30.82	25.16	-31.78	-108.84	-77.06
Quercus pubescens	15	7.48	31.19	23.71	-33.04	-99.75	-66.71
Quercus pubescens	15	8.78	31.32	22.53	-32.05	-98.30	-66.25
Quercus pubescens	15	7.58	32.37	24.79	-32.65	-94.95	-62.30
Quercus pubescens	20	6.25	30.99	24.74	-37.48	-101.21	-63.73
Quercus pubescens	20	5.94	30.28	24.35	-37.69	-99.69	-62.00
Quercus pubescens	20	6.87	31.66	24.79	-38.43	-107.95	-69.52
Quercus pubescens	25	2.99			-31.42	-90.5	-59.08
Quercus pubescens	25	3.78	28.90	25.13	-27.61	-100.47	-72.86
Quercus pubescens	25	4.32	29.62	25.30	-32.66	-100.63	-67.97
Quercus pubescens	30	2.07	27.89	25.82	-32.82	-91.78	-58.96
Quercus pubescens	30	2.89	28.07	25.19	-32.28	-89.63	-57.35
Quercus pubescens	30	1.47	27.81	26.33	-35.80	-95.12	-59.32
Quercus pubescens	35	3.45	28.10	24.65	-39.97	-84.64	-44.67
Quercus pubescens	35	0.53	27.29	26.76	-43.12	-94.95	-51.83
Quercus pubescens	35	2.28	27.22	24.93	-41.71	-88.04	-46.33
Quercus pubescens	40	2.77	30.10	27.33	-38.88	-77.40	-38.52
Quercus pubescens	40	2.41	26.79	24.38	-39.46	-77.68	-38.22
Quercus pubescens	40	0.11	25.03	24.92	-46.31	-77.16	-30.85
Salvia hispanica	10	3.86	31.79	27.93	-31.19	-115.52	-84.33
Salvia hispanica	10	4.48	32.19	27.71	-25.00	-114.82	-89.82
Salvia hispanica	10	4.35	32.18	27.82	-29.18	-136.73	-107.55
Salvia hispanica	15	5.05	32.58	27.53	-34.00	-124.71	-90.71
Salvia hispanica	15	5.53	32.98	27.45	-29.19	-107.62	-78.43
Salvia hispanica	15	6.66	33.89	27.23	-28.46	-127.15	-98.69
Salvia hispanica	20	2.91	31.10	28.19	-34.14	-111.53	-77.39
Salvia hispanica	20	2.06	31.05	28.99	-36.91	-98.32	-61.41
Salvia hispanica	20	1.51	29.93	28.42	-38.76	-112.22	-73.46
Salvia hispanica	25	0.08	28.80	28.72	-32.18	-139.78	-107.60
Salvia hispanica	25	1.10	28.99	27.89	-28.79	-124.99	-96.20
Salvia hispanica	25	0.62	28.20	27.58	-28.21	-122.37	-94.16
Salvia hispanica	30	0.68	26.96	26.28	-34.64	-121.25	-86.61
Salvia hispanica	30	0.38	26.75	26.37	-34.64	-104.44	-69.80
Salvia hispanica	30	0.43	26.46	26.02	-34.24	-119.16	-84.92
Salvia hispanica	35	7.83	23.27	15.44	-17.18	-122.33	-105.15
Salvia hispanica	35	-1.61	23.64	25.25	-39.63	-115.50	-75.87
Salvia hispanica	35	-1.55	23.93	25.48	-40.03	-112.04	-72.01
Salvia hispanica	40	-1.36	23.91	25.27	-39.66	-93.08	-53.42
Salvia hispanica	40	-3.20	22.40	25.60	-39.28	-98.05	-58.77
Salvia hispanica	40	-1.54	24.38	25.92	-40.07	-90.96	-50.89

Solanum cheesmaniae	10	2.13	33.30	31.17	-33.55	-137.17	-103.62
Solanum cheesmaniae	10	4.47	32.57	28.10	-25.74	-120.51	-94.77
Solanum cheesmaniae	10	2.99	33.88	30.88	-28.18	-123.74	-95.56
Solanum cheesmaniae	15	7.48	34.92	27.44	-26.19	-158.24	-132.05
Solanum cheesmaniae	15	5.63	34.51	28.88	-27.17	-152.67	-125.50
Solanum cheesmaniae	15	6.10	34.80	28.70	-27.04	-137.19	-110.15
Solanum cheesmaniae	20	5.74	33.93	28.19	-26.98	-171.35	-144.37
Solanum cheesmaniae	20	4.71	32.68	27.96	-29.24	-163.56	-134.32
Solanum cheesmaniae	20	3.74	33.53	29.79	-29.21	-161.94	-132.73
Solanum cheesmaniae	25	1.85	30.89	29.04	-25.19	-141.30	-116.11
Solanum cheesmaniae	25	2.34	30.66	28.32	-21.64	-168.91	-147.27
Solanum cheesmaniae	25	1.60	30.12	28.52	-20.77	-151.94	-131.17
Solanum cheesmaniae	30	1.33	28.59	27.26	-26.82	-119.63	-92.81
Solanum cheesmaniae	30	0.85	27.37	26.52	-29.73	-152.38	-122.65
Solanum cheesmaniae	30	1.25	28.27	27.02	-24.94	-132.18	-107.24
Solanum cheesmaniae	35	-1.44	24.61	26.05	-35.52	-132.35	-96.83
Solanum cheesmaniae	35	-1.68	24.44	26.12	-39.46	-119.93	-80.47
Solanum cheesmaniae	35	-2.58	23.88	26.46	-40.87	-133.11	-92.24
Solanum cheesmaniae	40	0.25	24.35	24.10	-28.59	-85.31	-56.72
Solanum cheesmaniae	40	-0.79	23.19	23.98	-30.07	-86.78	-56.71
Sorghum bicolor	10	5.16	32.29	27.12	-31.49	-98.28	-66.79
Sorghum bicolor	10	1.26	31.68	30.42	-35.06	-87.59	-52.53
Sorghum bicolor	10	3.29	32.86	29.57	-31.39	-104.12	-72.73
Sorghum bicolor	15	5.52	33.97	28.45	-32.28	-83.90	-51.62
Sorghum bicolor	15	3.92	30.16	26.25	-37.01	-91.17	-54.16
Sorghum bicolor	15	3.02	33.68	30.66	-39.83	-72.75	-32.92
Sorghum bicolor	20	10.25	32.84	22.59	-23.42	-87.26	-63.84
Sorghum bicolor	20	8.09	33.81	25.72	-25.76	-92.71	-66.95
Sorghum bicolor	20	4.19	34.19	30.00	-37.73	-123.64	-85.91
Sorghum bicolor	25	4.13	31.21	27.08	-23.59	-66.88	-43.29
Sorghum bicolor	25	5.25	31.94	26.69	-25.21	-66.94	-41.73
Sorghum bicolor	25	5.89	31.40	25.51	-13.48	-72.95	-59.47
Sorghum bicolor	30	3.55	30.89	27.34	-24.72	-57.95	-33.23
Sorghum bicolor	30	1.57	28.46	26.89	-27.90	-67.94	-40.04
Sorghum bicolor	30	-0.11	27.94	28.04	-37.35	-99.54	-62.19
Sorghum bicolor	35	-1.24	24.81	26.05	-41.67	-81.47	-39.80
Sorghum bicolor	35	-2.17	25.46	27.63	-41.87	-80.08	-38.21
Sorghum bicolor	35	1.43	26.30	24.87	-33.96	-100.60	-66.64
Sorghum bicolor	40	-1.63	25.26	26.89	-37.27	-87.10	-49.83
Sorghum bicolor	40	-1.16	26.19	27.35	-38.17	-57.49	-19.32
Sorghum bicolor	40	-1.32	25.22	26.54	-39.81	-96.41	-56.60

Supplementary Table 6: Comparison of the 34 tested models for δ^2H of leaf sugar (δ^2H_{LS} ‰) with their adjusted R^2 (and ranked by descending R^2), the deviance explained by each model: EHA25 = ε_{HA} at 25 °C (species-specific, ‰); d2HLW = δ^2H_{LW}, ‰, PercSta = Starch (% of NSC); temp = temperature [°C]; Anet = net assimilation rate (A_{net}, µmol CO_2 m^{-2} s^{-1}); Rdark = dark respiration rate (R_{dark}, µmol CO_2 m^{-2} s^{-1}), either as individual term (s(a)) or in interaction with another term (te(a,b)) and the selected degree of freedom (k). Generalized Cross-Validation (GCV) score: Lower GCV values indicate a better trade-off between model fit and complexity. Models were trained on 80% of the data and tested on the remaining 20%: Train RMSE (Root Mean Square Error) = the average magnitude of prediction errors on the training dataset; how well the model fits the data it was trained on. Test RMSE (Root Mean Square Error) = quantifies the prediction errors on the unseen test dataset, indicating the model's ability to generalize to new data. AIC (Akaike Information Criterion), evaluates the model's goodness of fit while penalizing complexity, with lower values indicating a better trade-off between accuracy and complexity. BIC (Bayesian Information Criterion): applies a stronger penalty for model complexity, favoring simpler models if they explain the data nearly as well.

Ranking by R^2	GAM: δ^2H_{LS} ~	adj. R^2	Deviance Explained (%)	GCV	ε_{HA25}	δ^2H_{LW}	R_{dark}	A_{net}	NSC (% dry mass)	Starch (% of NSC)	R_{dark}, A_{net}	temp, R_{dark}	temp, A_{net}	NSC (% dry mass), Starch (% of NSC)	Train RMSE	Test RMSE	AIC	BIC
1	s(avg_EHA_25, k = 4) + s(d2HLW, k = 4) + te(TotNSCmg100mg, PercSta, k = c(4, 4)) + te(temp, Anet, k = c(4, 4)) + te(temp, Rdark, k = c(4, 4))	0.884	90.7	163	***	n.s.						**	***	***	10.1	13.6	929	1015
2	s(avg_EHA_25, k = 4) + s(d2HLW, k = 4) + s(TotNSCmg100mg, k=4) + te(temp, Rdark, k = c(4, 4)) + te(temp, Anet, k = c(4, 4))	0.846	86.7	201	***	n.s.			***			*	***		12.7	15.0	955	1005
3	s(avg_EHA_25, k = 4) + s(d2HLW, k = 4) + s(TotNSCmg100mg, k=4) + s(Rdark, k = 4)	0.844	86.3	200	***	n.s.	n.s.		***				***		12.0	15.2	947	1003
4	s(avg_EHA_25, k = 4) + s(d2HLW, k = 4) + s(TotNSCmg100mg, k=4) + te(temp, Anet, k = c(4, 4))	0.844	86.3	198	***	n.s.			***				***		12.1	15.0	946	999
5	s(avg_EHA_25, k = 4) + s(TotNSCmg100mg, k=4) + te(temp, Rdark, k = c(4, 4)) + te(temp, Anet, k = c(4, 4))	0.843	86.4	202	***				***			*	***		12.7	15.4	956	1007
6	s(avg_EHA_25, k = 4) + s(TotNSCmg100mg, k=4) + te(temp, Anet, k = c(4, 4))	0.842	85.9	199	***				***				***		12.5	15.0	950	997
7	s(avg_EHA_25, k = 4) + s(TotNSCmg100mg, k=4) + s(Rdark, k = 4) + te(temp, Anet, k = c(4, 4))	0.841	85.9	201	***		n.s.		***				***		12.4	15.2	950	1000
8	s(avg_EHA_25, k = 4) + te(temp, Rdark, k = c(4, 4)) + te(temp, Anet, k = c(4, 4))	0.831	85.2	217	***							n.s.	***		13.1	15.0	963	1013
9	s(avg_EHA_25, k = 4) + s(d2HLW, k = 4) + te(temp, Rdark, k = c(4, 4)) + te(temp, Anet, k = c(4, 4))	0.831	85.3	218	***	n.s.						n.s.	***		13.0	15.0	964	1020
10	s(avg_EHA_25, k = 4) + s(Rdark, k = 4) + te(temp, Anet, k = c(4, 4))	0.828	84.7	217	***		n.s.						***		13.2	15.0	962	1010
11	s(avg_EHA_25, k = 4) + te(temp, Anet, k = c(4, 4))	0.828	84.6	215	***								***		13.2	15.1	960	1006
12	s(avg_EHA_25, k = 4) + s(d2HLW, k = 4) + s(Rdark, k = 4) + te(temp, Anet, k = c(4, 4))	0.827	84.7	220	***	n.s.	n.s.						***		13.0	15.0	963	1017
13	s(avg_EHA_25, k = 4) + s(d2HLW, k = 4) + te(temp, Anet, k = c(4, 4))	0.826	84.6	218	***	n.s.							***		13.0	15.2	962	1013
14	s(avg_EHA_25, k = 4) + s(d2HLW, k = 4) + s(Rdark, k = 4) + s(Anet, k = 4) + s(PercSta, k = 4)	0.774	78.8	269	***	n.s.	***	***		***					15.6	14.7	990	1022
15	s(avg_EHA_25, k = 4) + s(Rdark, k = 4) + s(Anet, k = 4) + s(PercSta, k = 4)	0.773	78.5	269	***		***	***		***					15.8	14.9	989	1016
16	s(avg_EHA_25, k = 4) + s(d2HLW, k = 4) + te(TotNSCmg100mg, PercSta, k = c(4, 4))	0.77	78.8	278	***	n.s.								***	15.5	14.5	993	1032
17	s(avg_EHA_25, k = 4) + s(d2HLW, k = 4) + s(PercSta, k = 4) + s(TotNSCmg100mg, k = 4)	0.754	76.9	292	***	n.s.			***	***					16.4	14.6	1001	1032
18	s(avg_EHA_25, k = 4) + s(PercSta, k = 4) + s(TotNSCmg100mg, k = 4)	0.747	75.8	297	***				***	***					16.9	14.5	1003	1027
19	s(avg_EHA_25, k = 4) + s(d2HLW, k = 4) + te(Rdark, Anet, k = 4)	0.73	75.1	328	***	n.s.					***				16.0	19.0	1003	1044
20	s(avg_EHA_25, k = 4) + s(d2HLW, k = 4) + s(PercSta, k = 4)	0.728	74.2	321	***	n.s.				***					17.6	14.4	1015	1043
21	s(avg_EHA_25, k = 4) + s(d2HLW, k = 4) + s(Rdark, k = 4) + s(TotNSCmg100mg, k = 4)	0.727	73.9	321	***	n.s.	***		***						16.5	18.6	1000	1029
22	s(avg_EHA_25, k = 4) + s(PercSta, k = 4)	0.726	73.7	319	***					***					17.9	14.2	1014	1035
23	s(avg_EHA_25, k = 4) + te(Rdark, Anet, k = 4)	0.722	74	333	***						***				16.5	19.3	1004	1038
24	s(avg_EHA_25, k = 4) + s(Rdark, k = 4) + s(TotNSCmg100mg, k = 4)	0.716	72.5	328	***		***		***						16.9	19.1	1001	1023
25	s(avg_EHA_25, k = 4) + s(d2HLW, k = 4) + s(Rdark, k = 4) + s(Anet, k = 4)	0.708	72.6	349	***	n.s.	***	***							17.2	19.5	1011	1042
26	s(avg_EHA_25, k = 4) + s(Rdark, k = 4) + s(Anet, k = 4)	0.704	71.8	348	***		***	***							17.3	19.6	1009	1035
27	s(avg_EHA_25, k = 4) + s(d2HLW, k = 4) + s(TotNSCmg100mg, k = 4)	0.678	69.1	376	***	*			***						18.5	18.4	1023	1047
28	s(avg_EHA_25, k = 4) + s(d2HLW, k = 4) + s(Rdark, k = 4)	0.67	68.4	386	***	n.s.	***								18.6	20.2	1023	1045
29	s(avg_EHA_25, k = 4) + s(Rdark, k = 4)	0.657	66.6	395	***		***								18.9	20.6	1023	1039
30	s(avg_EHA_25, k = 4) + s(d2HLW, k = 4) + s(Anet, k = 4)	0.656	67.4	406	***	*		***							19.1	18.8	1034	1062
31	s(avg_EHA_25, k = 4) + s(TotNSCmg100mg, k = 4)	0.655	66.3	395	***				***						19.3	18.9	1028	1044
32	s(avg_EHA_25, k = 4) + s(Anet, k = 4)	0.631	64.4	427	***			***							20.0	19.4	1039	1059
33	s(avg_EHA_25, k = 4) + s(d2HLW, k = 4)	0.612	62.7	450	***	**									20.6	19.4	1047	1067
34	s(avg_EHA_25, k = 4)	0.581	58.8	478	***										21.7	20.0	1053	1065

*** : p-value < 0.001

** : p-value < 0.01

* : p-value ≤ 0.05

n.s. (non-significant) : p-value > 0.05

Supplementary Table 7: Comparison of the 20 tested models for $\delta^{18}O$ of leaf sugar ($\delta^{18}O_{LS;}$ ‰) with their adjusted R^2 (and ranked by descending R^2), the deviance explained by each model: d18OLW = $\delta^{18}O_{LW,}$ ‰, PercSta = Starch (% of NSC); temp = temperature [°C]; Anet = net assimilation rate (A_{net}, µmol CO_2 m^{-2} s^{-1}); Rdark = dark respiration rate (R_{dark}, µmol CO_2 m^{-2} s^{-1}), either as individual term (s(a)) or in interaction with another term (te(a,b)) and the selected degree of freedom (k). Generalized Cross-Validation (GCV) score: Lower GCV values indicate a better trade-off between model fit and complexity. Models were trained on 80% of the data and tested on the remaining 20%: Train RMSE (Root Mean Square Error) = the average magnitude of prediction errors on the training dataset; how well the model fits the data it was trained on. Test RMSE (Root Mean Square Error) = quantifies the prediction errors on the unseen test dataset, indicating the model's ability to generalize to new data. AIC (Akaike Information Criterion), evaluates the model's goodness of fit while penalizing complexity, with lower values indicating a better trade-off between accuracy and complexity. BIC (Bayesian Information Criterion): applies a stronger penalty for model complexity, favoring simpler models if they explain the data nearly as well.

Ranking by R^2	GAM: $\delta^{18}O_{LS}$ ~	adj. R^2	Deviance Explained (%)	GCV	$\delta^{18}O_{LW}$	R_{dark}	A_{net}	NSC (% dry mass)	Starch (% of NSC)	R_{dark}, A_{net}	temp, R_{dark}	temp, A_{net}	NSC (% dry mass), Starch (% of NSC)	Train RMSE	Test RMSE	AIC	BIC
1	s(d18OLW, k = 4) + te(TotNSCmg100mg, PercSta, k = c(4, 4)) + te(temp, Anet, k = c(4, 4)) + te(temp, Rdark, k = c(4, 4))	0.876	89.2	1.81	***						*	***	***	1.2	1.2	411	469
2	s(d18OLW, k = 4) + s(TotNSCmg100mg, k=4) + s(Rdark, k = 4) + te(temp, Anet, k = c(4, 4))	0.868	88	1.87	***	**		***				***		1.3	1.2	412	459
3	s(d18OLW, k = 4) + s(TotNSCmg100mg, k=4) + te(temp, Rdark, k = c(4, 4)) + te(temp, Anet, k = c(4, 4))	0.865	87.5	1.86	***			***			***	**		1.3	1.1	415	457
4	s(d18OLW, k = 4) + s(TotNSCmg100mg, k=4) + te(temp, Anet, k = c(4, 4))	0.863	87.5	1.93	***			**				***		1.3	1.3	416	461
5	s(d18OLW, k = 4) + te(temp, Rdark, k = c(4, 4)) + te(temp, Anet, k = c(4, 4))	0.857	87.2	2.03	***						***	**		1.3	1.3	423	461
6	s(d18OLW, k = 4) + s(Rdark, k = 4) + te(temp, Anet, k = c(4, 4))	0.853	86.7	2.08	***	n.s.						***		1.3	1.4	421	464
7	s(d18OLW, k = 4) + te(temp, Anet, k = c(4, 4))	0.853	86.5	2.06	***							***		1.3	1.4	419	460
8	s(d18OLW, k = 4) + te(temp, Rdark, k = c(4, 4))	0.842	85.1	2.16	***						***			1.4	1.4	424	457
9	s(d18OLW, k = 4) + s(Rdark, k = 4) + s(TotNSCmg100mg, k = 4)	0.805	81.2	2.60	***	***		***						1.5	1.5	447	474
10	s(d18OLW, k = 4) + s(Rdark, k = 4) + s(Anet, k = 4) + s(PercSta, k = 4)	0.756	76.9	3.30	***	***	n.s.		*					1.7	1.8	469	498
11	s(d18OLW, k = 4) + te(Rdark, Anet, k = 4)	0.751	76.5	3.36	***					***				1.7	1.9	468	497
12	s(d18OLW, k = 4) + s(Rdark, k = 4) + s(Anet, k = 4)	0.745	75.6	3.41	***	***	n.s.							1.7	1.8	471	495
13	s(d18OLW, k = 4) + s(Rdark, k = 4)	0.744	75.3	3.40	***	***								1.7	1.9	469	490
14	s(d18OLW, k = 4) + s(TotNSCmg100mg, k = 4)	0.67	68.1	4.38	***			***						2.0	2.0	503	524
15	s(d18OLW, k = 4) + s(PercSta, k = 4) + s(TotNSCmg100mg, k = 4)	0.667	68.1	4.45	***			**	n.s.					2.0	2.0	505	529
16	s(d18OLW, k = 4) + te(TotNSCmg100mg, PercSta, k = c(4, 4))	0.665	68.2	4.52	***								**	2.0	1.9	510	536
17	s(d18OLW, k = 4) + s(Anet, k = 4)	0.644	65.3	4.68	***		*							2.1	2.0	511	527
18	s(d18OLW, k = 4)	0.634	64	4.78	***									2.2	2.0	513	526
19	s(d18OLW, k = 4) + s(PercSta, k = 4)	0.634	64.3	4.82	***				n.s.					2.2	2.0	514	530

*** : p-value < 0.001

** : p-value < 0.01

* : p-value ≤ 0.05

n.s. (non-significant) : p-value > 0.05
